# Bowel urgency in ulcerative colitis: effect of baseline urgency and change in urgency in response to mirikizumab

**DOI:** 10.1186/s41687-025-00906-0

**Published:** 2025-07-01

**Authors:** David B. Clemow, Marla C. Dubinsky, Simin K. Baygani, Bruce E. Sands, Anthony Keohane, Silvio Danese, Stefan Schreiber, Alissa J. Walsh, Toshifumi Hibi, Theresa Hunter Gibble, Richard E. Moses, Simon P. L. Travis

**Affiliations:** 1 Eli Lilly and Company, Lilly Corporate Center, Indianapolis, IN 46285 USA; 2 Mount Sinai Hospital, New York, NY USA; 3 Dr. Henry D. Janowitz Division of Gastroenterology, Icahn School of Medicine at Mount Sinai, New York, NY USA; 4 HaaPACS GmbH, Statistics, Schriesheim, Germany; 5 IRCCS San Raffaele Hospital, Vita-Salute San Raffaele University, Milan, Italy; 6 University Hospital Schleswig-Holstein, Kiel, Germany; 7 Oxford University Hospitals NHS Foundation Trust, Oxford, UK; 8 Kitasato University, Kitasato Institute Hospital, Tokyo, Japan; 9 Kennedy Institute of Rheumatology, Translational Gastroenterology Unit, and Biomedical Research Centre, University of Oxford, Oxford, UK

**Keywords:** Mirikizumab, Bowel urgency, Urgency numeric rating scale, Ulcerative colitis, Efficacy

## Abstract

**Background:**

Mirikizumab has demonstrated efficacy in moderately to severely active ulcerative colitis. A 1–2-point change in Urgency Numeric Rating Scale (NRS) score can be meaningful for patients. In these post-hoc analyses, we evaluated the efficacy of mirikizumab compared to placebo by baseline Urgency NRS score groups (0–3, 4–6, and 7–10) and its effect on bowel urgency severity over time.

**Methodology:**

Urgency NRS was measured as a secondary outcome at baseline, week 12, and week 52. Bowel urgency improvement was assessed for patients who achieved and did not achieve multiple efficacy endpoints. Data were analyzed using Fisher’s exact test with nonresponder imputation.

**Results:**

At weeks 12 and 52, a significantly higher percentage of mirikizumab-treated patients achieved clinical response as well as clinical, endoscopic, and symptomatic remission compared to placebo-treated patients, regardless of baseline Urgency NRS score category (higher proportions versus placebo, delta 9%–45%). Improvement in Urgency NRS score category at weeks 12 and 52 for mirikizumab-treated patients was observed when other efficacy outcomes were achieved (13%–90%) and not achieved (12%–75%).

**Conclusions:**

A greater proportion of mirikizumab-treated patients with ulcerative colitis achieved symptomatic, clinical, and endoscopic remission endpoints compared to placebo-treated patients, regardless of baseline bowel urgency severity. After one year, bowel urgency was improved to a greater extent with mirikizumab than with placebo, even for patients who did not achieve other clinical outcomes. Small improvements in bowel urgency are associated with significant health-related quality-of-life improvements. Monitoring shifts in urgency severity over time using the Urgency NRS can aid in understanding patients’ treatment outcomes.

**Trial registration:**

LUCENT-1 (NCT03518086) Registered 04 May 2018 https://clinicaltrials.gov/study/NCT03518086. LUCENT-2 (NCT03524092) Registered 10 May 2018 https://clinicaltrials.gov/study/NCT03524092.

**Supplementary information:**

The online version contains supplementary material available at 10.1186/s41687-025-00906-0.

## Abstract (de)

For patients with ulcerative colitis and different levels of bowel urgency severity at baseline, mirikizumab improved bowel urgency more than placebo, even for those who did not achieve full clinical benefit. Small improvements in bowel urgency are associated with significant health-related quality of life improvements, so assessing bowel urgency improvements over time aids in understanding patients’ treatment outcomes.

## Background

Bowel urgency, the sudden and immediate need for a bowel movement, is a distinct symptom of ulcerative colitis (UC), along with more commonly assessed symptoms such as rectal bleeding and stool frequency [1, 2]. Bowel urgency has gained increasing attention as patients have identified it as an important symptom [3–7] and the one they most want improved with treatment [8–10], given its profound impact on their health-related quality of life [11–14].

Most instruments assessing UC disease activity, including the modified Mayo score, do not assess bowel urgency [15]. The Urgency Numeric Rating Scale (NRS) was designed to assess change in bowel urgency severity over time for patients with UC [16, 17]. The Urgency NRS captures bowel urgency severity on a 0–10 scale, with higher scores indicating greater bowel urgency. The Urgency NRS distinguishes levels of severity instead of a categorical “yes/no” response option.

Mirikizumab is an anti-interleukin-23 p19 antibody approved for the treatment of moderately to severely active UC and under development for Crohn’s disease [18, 19]. It has demonstrated efficacy and safety in patients with moderately to severely active UC, including improvement in bowel urgency, in the phase 3 induction LUCENT-1 and maintenance LUCENT-2 trials [20].

These post hoc analyses aimed to (1) evaluate the efficacy of mirikizumab induction and maintenance treatment compared to placebo among patients with different levels of bowel urgency severity at baseline, as measured by the Urgency NRS; (2) determine whether the achievement of efficacy endpoints was associated with bowel urgency improvement; (3) ascertain the magnitude of bowel urgency shifts in severity over time using the Urgency NRS; and (4) examine the clinical relevance of small shifts in bowel urgency improvement for patients.

## Methods

### Study designs

LUCENT-1 (ClinicalTrials.gov identifier: NCT03518086) was a 12-week, multinational, phase 3, randomized, parallel-arm, double-blind, placebo-controlled induction trial of mirikizumab in patients with moderately to severely active UC. LUCENT-2 (ClinicalTrials.gov identifier: NCT03524092) was a 40-week, multinational, phase 3, randomized, double-blind, withdrawal maintenance study in patients who responded to mirikizumab induction therapy in LUCENT-1. The design and results of both studies have been previously described in detail [20].

These clinical studies were performed in compliance with the International Council for Harmonisation of Technical Requirements for Pharmaceuticals for Human Use Good Clinical Practice guidelines [21]. All patients provided written informed consent, and the study protocol was approved by each site’s institutional review board or ethics committee before the start of both studies.

Briefly, the LUCENT-1 induction study included patients aged 18 to 80 years with a UC diagnosis of at least 3 months before baseline and moderately to severely active disease (based on a modified Mayo score of 4–9). They were randomized 3:1 to receive mirikizumab 300 mg or placebo, administered by intravenous infusion every 4 weeks. Patients were stratified based on previous failure or intolerance to biologic therapy (bio-failed status; yes/no), baseline corticosteroid use (yes/no), baseline disease activity (modified Mayo score of 4–6 vs 7–9), and geographic region (North America/Europe/other).

In LUCENT-2, patients who received mirikizumab and met the clinical response criteria at week 12 in the induction study were rerandomized 2:1 to receive mirikizumab 200 mg or placebo administered by subcutaneous injection every 4 weeks for 40 weeks. Clinical response was defined as a ≥ 2-point decrease in the modified Mayo score, with a ≥ 30% reduction from baseline, plus a ≥ 1-point decrease from baseline or a score of 0 or 1 in the rectal bleeding subscore. At LUCENT-2 randomization, patients were stratified by bio-failed status, region, corticosteroid use at induction baseline, and clinical remission status at week 12.

### Study population

All the current analyses were performed on the modified intention-to-treat population, which included all patients who received any study treatment during the study and excluded those impacted by an electronic clinical outcome assessment transcription error in Poland and Turkey [20], regardless of whether they followed the protocol.

Patients with a clinical response to mirikizumab therapy at the end of LUCENT-1 (week 12) were rerandomized to either mirikizumab or placebo for the maintenance period (weeks 12 through 52). Hence, for the week 52 analysis, those receiving mirikizumab had been treated for up to 52 weeks, while the placebo patients had responded after receiving mirikizumab for 12 weeks, and then received placebo afterwards.

### Efficacy endpoints

The Urgency NRS is a validated, patient-reported single item assessing the severity of bowel urgency [22]. The scale is rated from 0 (no urgency) to 10 (worst possible urgency) over the previous 24 hours and averaged over the previous 7 days. Psychometric evaluation has demonstrated that a ≥ 3-point change in the Urgency NRS score is a clinically meaningful improvement, and a score of 0 or 1 represents bowel urgency remission [16].

Patients used a daily electronic diary to record their Urgency NRS score. Weekly scores were calculated as the average of available daily entries over a 7-day period (rounded to the nearest integer). At least 4 days of data were required, or the patient’s data were considered missing for that week.

The following Urgency NRS score groups were used to assess bowel urgency severity at baseline: Urgency NRS scores of 0–3, 4–6, and 7–10. The results were similar across the clinical endpoints assessed; therefore, clinical remission was used as the efficacy outcome (highlighted in the figures within this article; the other endpoints are noted with the relevant data provided in the Supplementary Material).

The following endpoints were assessed for the proportion of patients who achieved an endpoint by baseline Urgency NRS subgroup at weeks 12 and 52 for mirikizumab versus placebo:

- Alternative clinical remission: modified Mayo score stool frequency subscore of 0 or 1, rectal bleeding subscore of 0, and endoscopic subscore of 0 or 1 (excluding friability).
- Change from baseline in bowel urgency: single-point or categorical group (0–3, 4–6, or 7–10) score changes on the 11-point Urgency NRS from 0 (no urgency) to 10 (worst possible urgency).
- Clinically meaningful improvement in bowel urgency: ≥ 3-point decrease from baseline in the Urgency NRS score for patients with an Urgency NRS score ≥ 3 at induction baseline.
- Bowel urgency remission: Urgency NRS score of 0 or 1.
- Clinical response: ≥ 2-point and ≥ 30% decrease in the modified Mayo score from baseline, and rectal bleeding subscore of 0 or 1 or ≥ 1-point decrease in the modified Mayo score from baseline.
- Clinical remission: stool frequency subscore of 0 or 1 with ≥ 1-point decrease in the modified Mayo score from baseline, rectal bleeding subscore of 0, and endoscopic subscore of 0 or 1 (excluding friability).
- Corticosteroid-free remission (week 52): clinical remission at week 40 of LUCENT-2, symptomatic remission at week 28, and no corticosteroid use for ≥ 12 weeks prior to week 40.
- Endoscopic remission: endoscopic subscore of 0 or 1 (excluding friability).
- Endoscopic response: ≥ 1-point decrease in the endoscopic subscore compared to baseline.
- Histologic-endoscopic mucosal remission: Geboes score ≤ 2B.0 plus endoscopic subscore of 0 or 1 (excluding friability), histologic remission with resolution of neutrophils (defined using Geboes score ≤ 2B.0), and Geboes subscore of 0 for grades 2b (lamina propria neutrophils), 3 (neutrophils in epithelium), 4 (crypt destruction), and 5 (erosion or ulceration).
- Inflammatory Bowel Disease Questionnaire (IBDQ) remission: IBDQ total score ≥ 170.
- Rectal bleeding remission: rectal bleeding subscore of 0.
- Stool frequency remission: stool frequency subscore of 0 or 1 with ≥ 1-point decrease from baseline.
- Symptomatic remission: stool frequency subscore of 0 or 1 with ≥ 1-point decrease in the modified Mayo score from baseline; rectal bleeding subscore of 0.
- Symptomatic response: ≥ 30% decrease from baseline in the composite clinical endpoint of the sum of stool frequency and rectal bleeding subscores.

For the proportion of patients who shifted from one Urgency NRS score group (0–3, 4–6, or 7–10) to another, the following endpoints were assessed: clinically meaningful improvement in bowel urgency, bowel urgency remission, clinical remission, clinical response, endoscopic remission, histologic-endoscopic mucosal remission, IBDQ remission, and symptomatic remission. Corticosteroid-free remission was also assessed (at week 52 only). Endpoints were assessed at weeks 12 and 52. The treatment groups were mirikizumab versus placebo, and the subgroups included patients who achieved the endpoint of interest versus those who did not.

### Statistical analyses

Analyses used logistic regression with treatment, subgroup, and treatment-by-subgroup interaction, as well as prior biologic or tofacitinib failure (yes/no), baseline corticosteroid use (yes/no), baseline disease activity (modified Mayo score of 4–6 or 7–9), and region (North America/Europe/other). Urgency NRS score group at baseline was the subgroup variable used.

Fisher’s exact test was performed with nonresponder imputation by patient scores on the Urgency NRS, grouped as 0–3 (low), 4–6 (intermediate), and 7–10 (high). Confidence intervals were constructed using the asymptotic method, without continuity correction. While Fisher’s Exact test would produce *P* values for comparisons with small patient numbers, to avoid concerns of analytical robustness, it was determined a priori in the analysis plan that “not applicable” would be displayed for *P* values associated for comparisons where the number of patients in a subgroup was < 10% of the overall population.

Nonresponder imputation was used to handle missing binary/categorical data. Patients who discontinued treatment or had missing endpoint assessments were treated as nonresponders. A modified baseline observation carried forward approach was used to impute missing continuous data, such as Urgency NRS scores. There were no adjustments made for multiplicity.

## Results

### Baseline demographics and clinical characteristics

Baseline demographic and clinical characteristics are shown in Table 1 by baseline Urgency NRS score at LUCENT-1 baseline for the LUCENT-1 induction and LUCENT-2 maintenance populations evaluated in the current analyses. The maintenance data only include patients who were responders to mirikizumab induction therapy from LUCENT-1; the placebo group in LUCENT-2 comprised responders to mirikizumab induction therapy in LUCENT-1 who were rerandomized to placebo in LUCENT-2 (“mirikizumab-to-placebo” patients) (see Patient disposition in Supplementary Figure 1). The demographics and clinical characteristics of patients were mostly similar across the baseline Urgency NRS score groups. However, for patients in the Urgency NRS 7–10 score group, there were slightly fewer males and more Americans and Europeans than those from other geographic regions. Disease severity—as measured by the mean modified Mayo score, severity category, and endoscopic subscore—and prior biologic failure at baseline were somewhat greater for patients in the Urgency NRS 7–10 group compared with those in the 4–6 group and for those in the Urgency NRS 4–6 group compared with those in the 0–3 group. Baseline corticosteroid use was highest for patients in the Urgency NRS 7–10 group followed by those in the Urgency NRS 0–3 and then 4–6 groups.

**Table 1:** Baseline patient demographics and clinical characteristics

	**LUCENT-1**			LUCENT-2^a^			
	Baseline Urgency NRS score			Baseline Urgency NRS score			
	0–3 **(*N*** **= 149)**	4–6 **(*N*** **= 437)**	7–10 (*N* = 576)	0–3 (*N* = 70)	4–6 (*N* = 215)	7–10 (*N* = 259)	
Age, years, mean (SD)	45.0 (15.3)	43.4 (14.6)	41.2 (12.9)	45.3 (15.1)	43.7 (14.8)	41.1 (12.4)	
Male sex, *n* (%)	95 (63.8)	273 (62.5)	327 (56.8)	45 (64.3)	132 (61.4)	141 (54.4)	
Geographic region, *n* (%)							
North America	20 (13.4)	59 (13.5)	106 (18.4)	8 (11.4)	25 (11.6)	41 (15.8)	
Europe	50 (33.6)	144 (33.0)	228 (39.6)	22 (31.4)	70 (32.6)	101 (39.0)	
Other	79 (53.0)	234 (53.5)	242 (42.0)	40 (57.1)	120 (55.8)	117 (45.2)	
Duration of UC, years, mean (SD)	7.3 (6.6)	7.1 (6.8)	7.0 (6.9)	6.7 (5.1)	6.7 (6.4)	6.9 (7.2)	
Disease location, *n* (%)							
Left-sided colitis	88 (59.1)	275 (62.9)	369 (64.2)	39 (55.7)	141 (65.6)	173 (66.8)	
Pancolitis	60 (40.3)	159 (36.4)	202 (35.1)	30 (42.9)	73 (34.0)	84 (32.4)	
Modified Mayo score, mean (SD)	5.6 (1.2)	6.3 (1.2)	6.9 (1.3)	5.6 (1.2)	6.3 (1.2)	7.0 (1.2)	
Modified Mayo score category, *n* (%)							
Moderate (score of 4–6)	112 (75.7)	235 (53.8)	195 (33.9)	53 (75.7)	122 (56.7)	83 (32.0)	
Severe (score of 7–9)	36 (24.3)	201 (46.0)	381 (66.1)	17 (24.3)	93 (43.3)	176 (68.0)	
ES, severe (score of 3)	85 (57.4)	288 (65.9)	401 (69.6)	32 (45.7)	134 (62.3)	175 (67.6)	
Corticosteroid use, *n* (%)	59 (39.6)	154 (35.2)	251 (43.6)	27 (38.6)	67 (31.2)	109 (42.1)	
Immunomodulator use, *n* (%)	40 (26.8)	114 (26.1)	126 (21.9)	18 (25.7)	44 (20.5)	55 (21.2)	
Prior biologic failure, *n* (%)^b^	50 (33.6)	172 (39.4)	257 (44.6)	15 (21.4)	78 (36.3)	99 (38.2)	

^a^For the week 52 analyses, mirikizumab-treated patients were those who had responded to mirikizumab induction therapy at week 12 and were rerandomized to continue mirikizumab or switch to placebo during maintenance treatment

^b^Prior biologic failure was defined as prior inadequate response, loss of response, or intolerance to biologic therapy or the Janus kinase inhibitor tofacitinib

*Abbreviations* ES, endoscopic subscore; *N*, number of patients in the analysis population; *n*, number of patients in each category; NRS, Numeric Rating Scale; SD, standard deviation; UC, ulcerative colitis

Supplementary Figure 2 shows the LUCENT-1 induction study Urgency NRS scores at baseline (week 0) for patients who received mirikizumab in LUCENT-1 and then mirikizumab or placebo in LUCENT-2. The Urgency NRS score at this time point was considered the baseline score for all analyses.

### Efficacy based on baseline Urgency NRS score

**Fig. 1:**
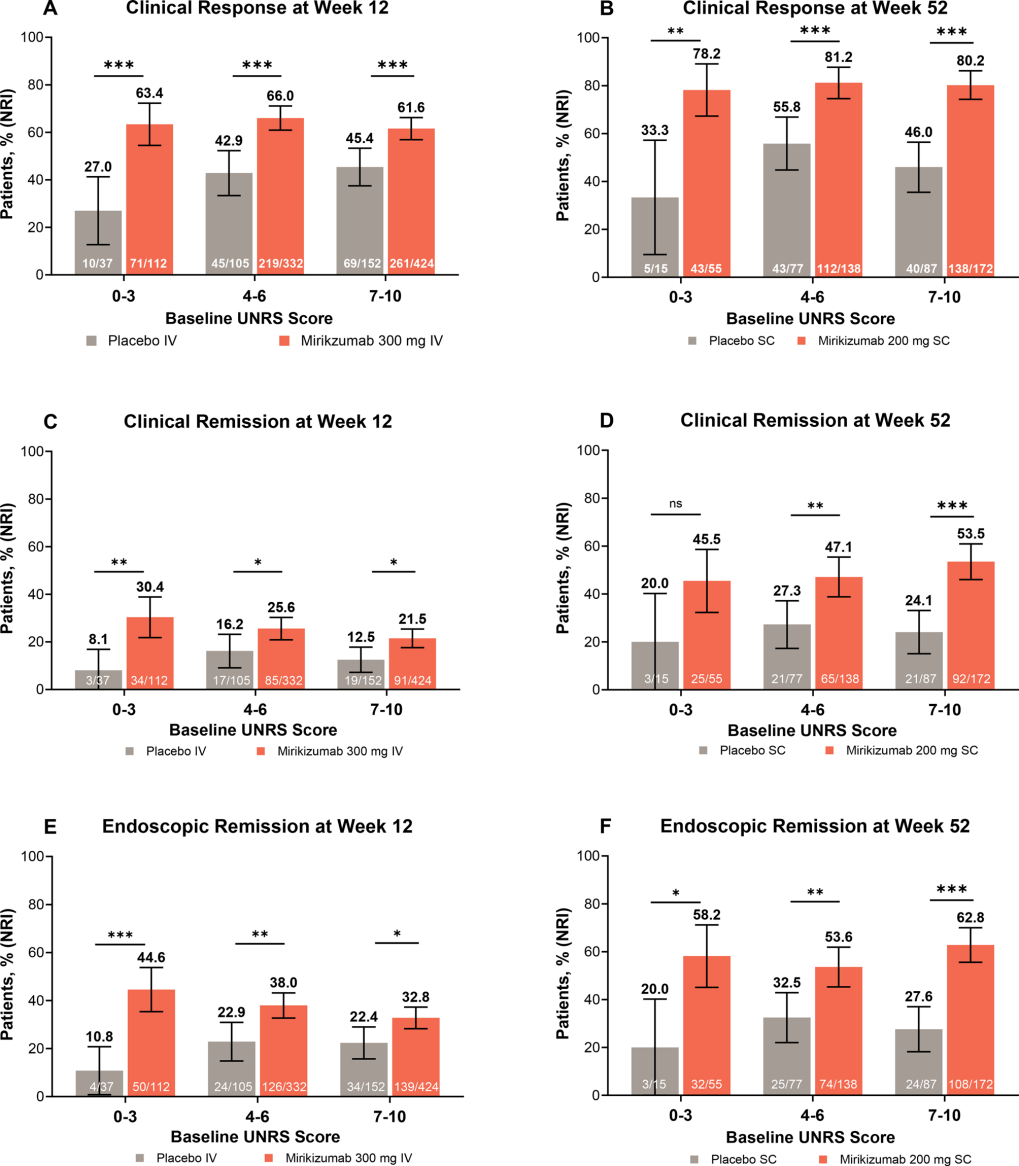
Clinical response, clinical remission, or endoscopic remission at weeks 12 and 52. Using NRI, data are shown as the percentage (95% CI) of patients in the modified intention-to-treat population meeting the criteria for clinical response at **(A)** week 12 and **(B)** week 52, clinical remission at **(C)** week 12 and **(D)** week 52, or endoscopic remission at **(E)** week 12 and **(F)** week 52, for patients who received (A, C, E) mirikizumab versus placebo or (B, D, F) mirikizumab versus mirikizumab-to-placebo by Urgency NRS score at induction baseline. *P < 0.05; **P < 0.01; ***P < 0.001 vs PBO. Week 12: PBO, *N* = 294; MIRI, *N* = 868. Week 52: PBO, *N* = 179; MIRI, *N* = 365. See the *Efficacy Endpoints* section for definitions. Abbreviations: CI, confidence interval; IV, intravenous; MIRI, mirikizumab; *N*, number of patients in the analysis population or the number of patients in each Urgency NRS score grouping; *n*, number of patients within analyzed group achieving the endpoint of interest; NRI, nonresponder imputation; NRS, Numeric Rating Scale; ns, not significant; SC, subcutaneous; UNRS, Urgency Numeric Rating Scale

To assess the relationship between baseline Urgency NRS score and response to mirikizumab, various measures of treatment response (see *Efficacy Endpoints* section) were evaluated at weeks 12 and 52 by baseline Urgency NRS score group. A significantly greater improvement in clinical response, clinical remission (with one exception), and endoscopic remission was observed at week 12 for patients who received mirikizumab versus placebo. At week 52, patients who continued mirikizumab showed significantly greater improvement compared to those who were rerandomized to placebo for each baseline Urgency NRS score group (Fig. 1; Supplementary Video 1). For example, the proportion of clinical responders to mirikizumab ranged from 62% (baseline Urgency NRS 7–10 group) to 66% (baseline Urgency NRS 4–6 group) at week 12, and maintenance of clinical response was seen in 78% (baseline Urgency NRS 0–3 group) to 81% (baseline Urgency NRS 4–6 group) of patients at week 52.

**Fig. 2:**
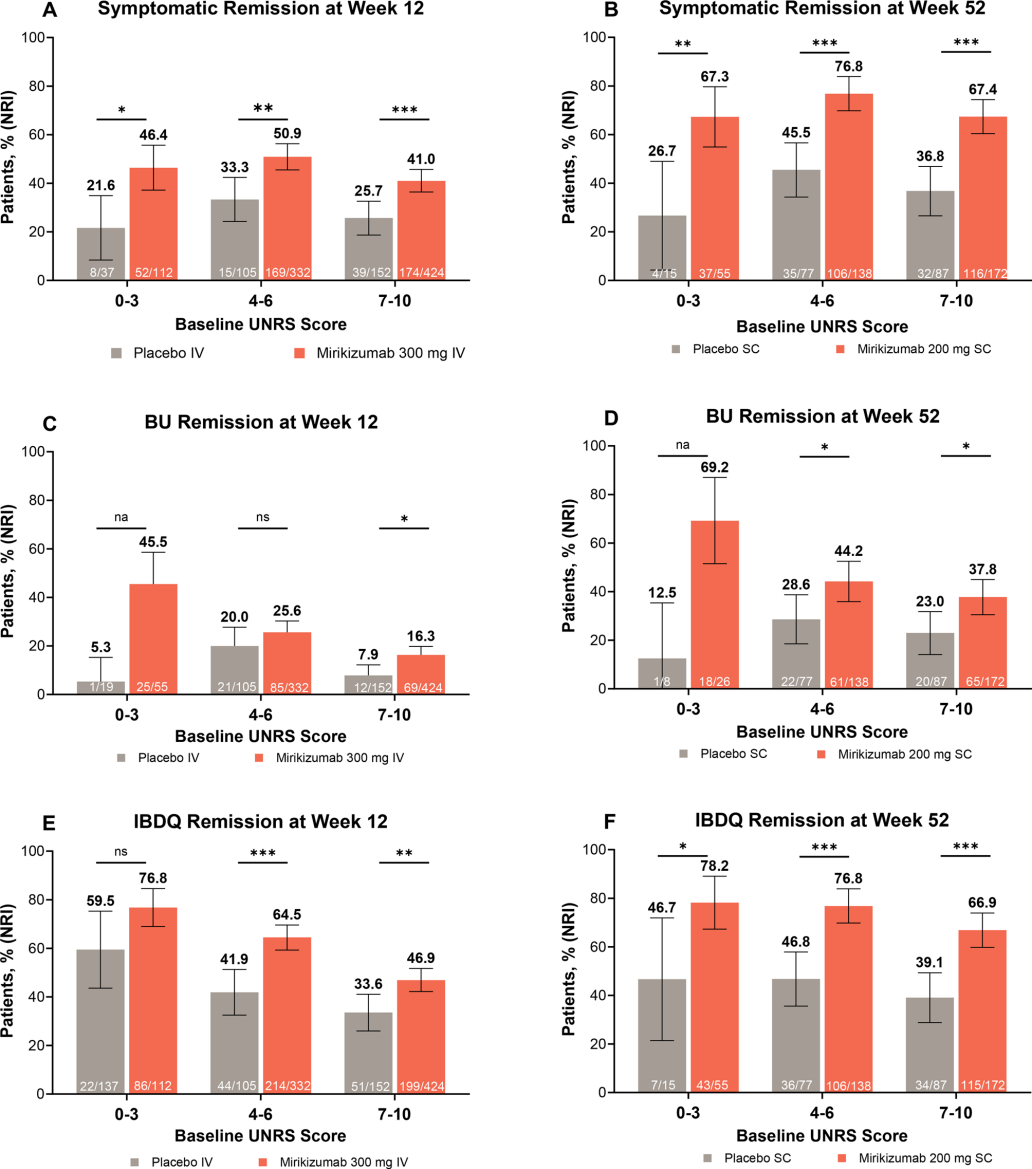
**(A, B)** Symptomatic remission, **(C, D)** BU remission, or **(E, F)** IBDQ remission at weeks 12 and 52. Using NRI, data are shown as the percentage (95% CI) of patients in the modified intention-to-treat population meeting the criteria for symptomatic remission at (**A**) week 12 and (**B**) week 52, BU remission at (**C**) week 12 and (**D**) week 52, or IBDQ remission at (**E**) week 12 and (**F**) week 52, for patients who received (**A**, **C**, **E**) mirikizumab versus placebo or (**B**, **D**, **F**) mirikizumab versus mirikizumab-to-placebo by Urgency NRS score at induction baseline. *P < 0.05; **P < 0.01; ***P < 0.001 vs PBO. Week 12: Placebo, *N* = 294; MIRI, *N* = 868. Week 52: PBO, *N* = 179; MIRI, *N* = 365. See the *Efficacy Endpoints* section for definitions. The number of patients for BU remission is lower because this endpoint was only calculated for patients with an Urgency NRS score ≥ 3 at baseline. Abbreviations: BU, bowel urgency; CI, confidence interval; IBDQ, Inflammatory Bowel Disease Questionnaire; IV, intravenous; MIRI, mirikizumab; *N*, number of patients in the analysis population OR the number of patients in each Urgency NRS score grouping; *n*, number of patients within analyzed group achieving the endpoint of interest; na, not applicable (due to small N); NRI, nonresponder imputation; NRS, Numeric Rating Scale; ns, not significant; SC, subcutaneous; UNRS, Urgency Numeric Rating Scale

Similarly, mirikizumab demonstrated significantly greater improvements in symptomatic remission, bowel urgency remission, and IBDQ remission compared to placebo at week 12 and mirikizumab-to-placebo at week 52 across all three baseline Urgency NRS score groups (Fig. 2), with minor exceptions (week 12: category 4–6, bowel urgency remission; category 0–3, IBDQ). The proportion of mirikizumab symptomatic remitters ranged from 41% to 51% at week 12 and 67% to 77% at week 52.

Similar findings were also observed for alternative clinical remission, clinically meaningful improvement in bowel urgency, corticosteroid-free remission, endoscopic response, histologic-endoscopic mucosal remission, rectal bleeding remission, stool frequency remission, and symptomatic response (Supplementary Table 1).

### Severity shifts in Urgency NRS score from baseline

Shift analyses were performed by both Urgency NRS score group and Urgency NRS single-point or greater changes in bowel urgency to identify the magnitude of changes in severity associated with improvements following mirikizumab treatment (Fig. 1; Supplementary Video 1). Clinical remission data are shown as an example because of their clinical importance and because they include elements of symptomatic and endoscopic remission. Similar results to those for clinical remission were observed for the other endpoints assessed, unless otherwise noted (Supplementary Table 2).

**Fig. 3:**
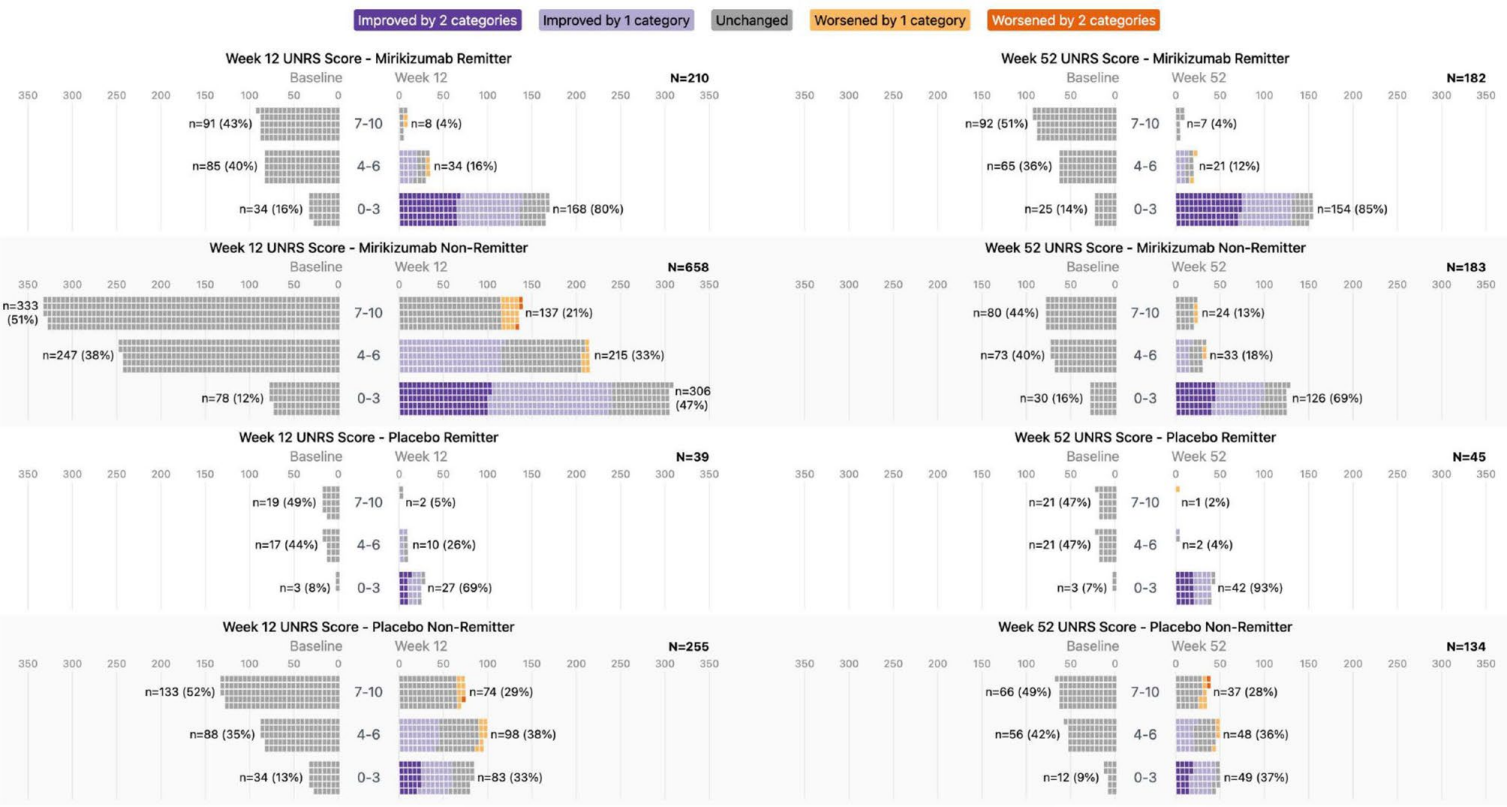
Shift in Urgency NRS score group from induction baseline to weeks 12 and 52 by treatment and clinical remission status. Purple indicates improvement by 2 group levels, light purple indicates improvement by 1 group level, gray indicates no change, light orange indicates worsening by 1 level, and dark orange indicates worsening by 2 levels. Patients at week 52 were responders to mirikizumab induction therapy at week 12 who were rerandomized to mirikizumab or placebo (treatment withdrawal). Abbreviations: *N*, number of patients in the analysis population OR the number of patients in each Urgency NRS score grouping; *n*, number of patients within analyzed group achieving the endpoint of interest; NRS, Numeric Rating Scale; UNRS, Urgency Numeric Rating Scale

Figure 3 provides shift data for the Urgency NRS score groups from induction baseline to weeks 12 and 52 by treatment and clinical remission status. Among mirikizumab-treated patients who achieved clinical remission at week 12, the proportion in the Urgency NRS 0–3 score group was 16% at baseline and 80% at week 12. Similarly, among mirikizumab induction responders who continued on mirikizumab and achieved clinical remission at week 52, 14% and 85% were in the Urgency NRS 0–3 score group at induction baseline and at week 52, respectively. Similar shifts in Urgency NRS scores occurred among induction placebo-treated patients (week 12) and mirikizumab-to-placebo patients (week 52) who achieved clinical remission. Among clinical nonremitters at week 12, 47% of mirikizumab-treated patients and 33% of placebo-treated patients had an Urgency NRS score of 0–3 at week 12 compared to 12% and 13%, respectively, at baseline. For clinical nonremitters at week 52, 69% who received mirikizumab and 37% who had switched to placebo at week 12 had an Urgency NRS score of 0–3. During maintenance treatment, the difference between patients who received mirikizumab and those who switched to placebo was largest among those who did not achieve clinical remission.

Supplementary Table 3 shows the shift data for Urgency NRS scores from induction baseline to weeks 12 and 52 for mirikizumab- and placebo-treated patients who did and did not achieve clinical remission by induction baseline Urgency NRS score group. Mirikizumab generally improved Urgency NRS scores (reduced severity) to a greater extent than did placebo at weeks 12 and 52 across all efficacy endpoints assessed, regardless of baseline Urgency NRS score or whether the clinical endpoint of interest was achieved or not (Fig. 1; Supplementary Video 1).

**Fig. 4:**
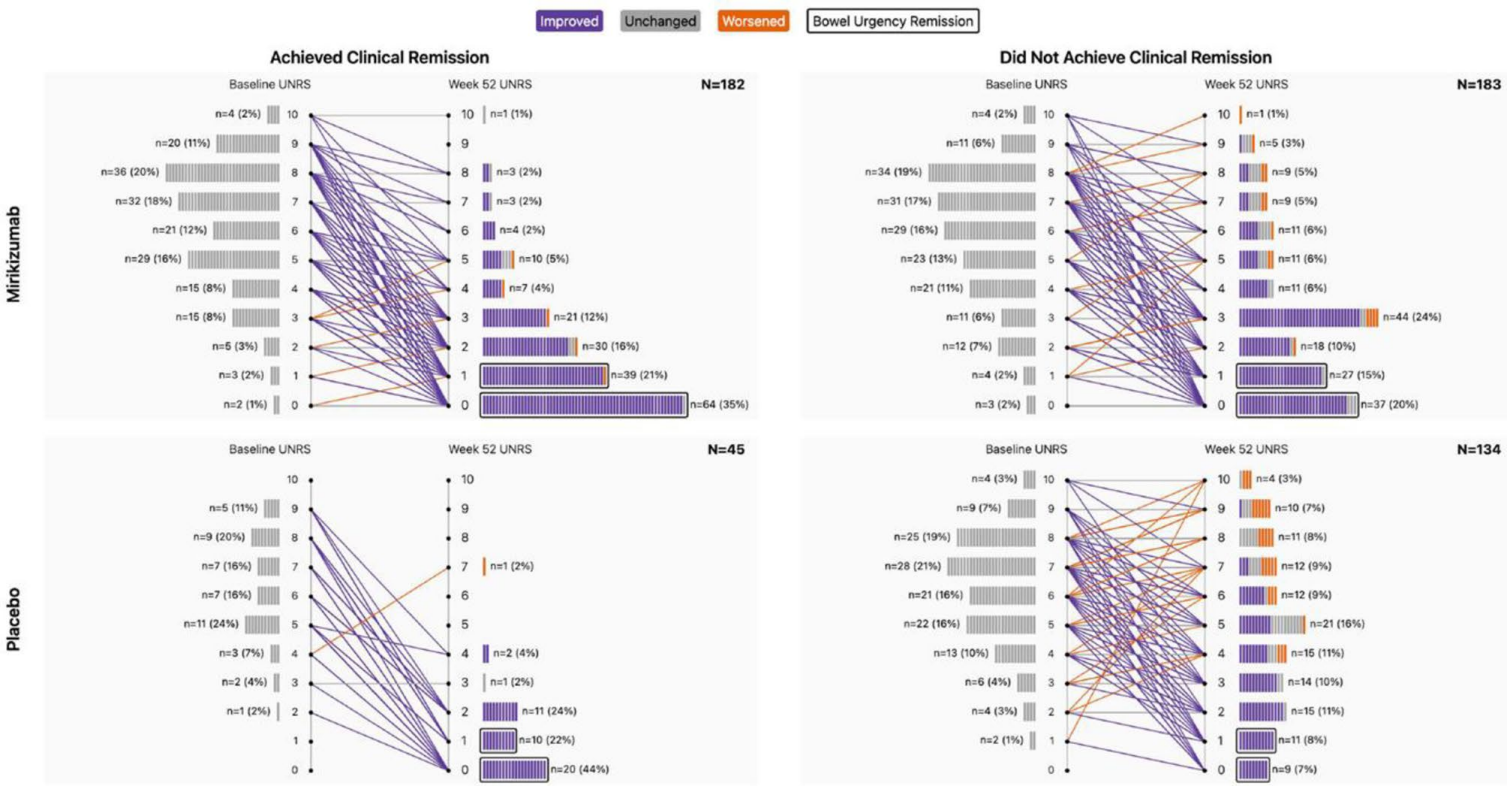
Shift in Urgency NRS score from induction baseline to week 52 by treatment and clinical remission status. Purple indicates improvement, gray indicates no change, and orange indicates worsening. An Urgency NRS score of 0 or 1 is considered bowel urgency remission and is noted with a black outline. Patients at week 52 were responders to mirikizumab induction therapy at week 12 who were rerandomized to mirikizumab or placebo (treatment withdrawal). Abbreviations: N, number of patients in the analysis population OR the number of patients in each Urgency NRS score grouping; n, number of patients within analyzed group achieving the endpoint of interest; NRS, Urgency Numeric Rating Scale; UNRS, Urgency Numeric Rating Scale

Figure 4 shows the shift in the Urgency NRS score from induction baseline to week 52 by treatment and clinical remission status. Overall, 168 of 182 (92%) mirikizumab-treated patients who achieved clinical remission had improved Urgency NRS scores at week 52, while 142 of 183 (78%) mirikizumab-treated patients who did not meet the clinical remission criteria had improved Urgency NRS scores at week 52. Supplementary Fig. 3 shows each Urgency NRS baseline score shift from Fig. 4 visualized individually to illustrate the shift in individual-point Urgency NRS score change.

Supplementary Table 4 shows the data for shift in individual-point Urgency NRS scores from induction baseline to week 52 for mirikizumab- and placebo-treated patients who did and did not achieve clinical remission by individual-point Urgency NRS score at induction baseline. Individual-point Urgency NRS score shifts illustrate that score shifts across all efficacy endpoints assessed were generally distributed across lower scores at week 52 compared with baseline, regardless of baseline Urgency NRS score (urgency severity; Supplementary Video 1). Across endpoints studied, greater improvement shifts in Urgency NRS score were observed for mirikizumab-treated patients compared with placebo-treated patients, and for patients achieving the clinical endpoint of interest compared with those who did not. However, for mirikizumab-treated patients, improved shifts in Urgency NRS were observed even when the clinical endpoint of interest was not achieved. Supplementary Fig. 4 provides an example to illustrate this finding, and Fig. 3 illustrates the magnitude of Urgency NRS score shift based on treatment and endpoint achievement status, using clinical remission as the endpoint. Similar trends were observed across the clinical endpoints assessed.

Supplementary Figure 4A provides an example pulled from Supplementary Table 4 to demonstrate individual-point Urgency NRS score shifts from induction baseline to week 52 for mirikizumab-treated patients. The example illustrates shifts from induction baseline for mirikizumab-treated patients who achieved clinical remission and had a baseline Urgency NRS score of 6: 14.3% improved by 1–2 points, which is considered clinically important to patients [16]. Others had improvements of 3–6 points, which are all clinically meaningful improvements [16]. In total, 57.1% shifted to bowel urgency remission (Urgency NRS score of 0 or 1). Supplementary Figure 4B illustrates how greater improvements in Urgency NRS score occurred with mirikizumab versus placebo and how these improvements were better for both mirikizumab- and placebo-treated patients when the clinical endpoint (clinical remission in this example) was achieved versus when it was not achieved (Supplementary Video 1).

For clinically meaningful improvement in bowel urgency, bowel urgency remission, clinical response, corticosteroid-free remission (week 52 only), endoscopic remission, histologic-endoscopic mucosal remission, IBDQ remission, and symptomatic remission, similar findings were observed in the proportion of patients who shifted from one baseline Urgency NRS score to another at weeks 12 and 52, as those observed for clinical remission (Supplementary Table 2).

## Discussion

These post hoc analyses aimed to evaluate the efficacy of mirikizumab induction and maintenance treatment among patients with UC who had different degrees of bowel urgency at baseline. Additional goals were to evaluate treatment-driven shifts in bowel urgency severity over time and determine the frequency of smaller changes that might have clinical relevance. Results from the present analyses show that the Urgency NRS provides a dynamic measure to quantify bowel urgency, which is important given that urgency is a UC symptom that can occur independently of the typical symptoms related to mucosal inflammation, such as blood in the stool and increased stool frequency [23]. Patients in clinical remission often have clinically meaningful improvement or remission of bowel urgency. However, bowel urgency may also persist even when patients’ symptoms are in clinical remission, which is important because improvement in bowel urgency is associated with better clinical outcomes compared to no improvement in bowel urgency [15]. Mirikizumab generally improved Urgency NRS scores to a greater extent than placebo at weeks 12 and 52 (in mirikizumab responders rerandomized to placebo at week 12) across all efficacy endpoints assessed, regardless of baseline Urgency NRS score or whether the clinical endpoint of interest was achieved. These data suggest that mirikizumab may be a valuable treatment option for patients with UC struggling with bowel urgency.

The mechanisms of bowel urgency for patients with UC may be multifactorial. In addition to the underlying mucosal inflammation due to overactivation of the immune response (increased inflammatory cytokines such as interleukin-23) causing impaired barrier function of the intestinal wall [24, 25], factors may include inflammatory changes of the rectum, hypersensitivity of the rectum, rectal contractile response/spasms, increased reactivity to rectal distension, increased stool influx due to impaired colon function, and development of submucosal fibrosis associated with chronic inflammation causing decreased rectal wall compliance [26–32]. Thus, patients with bowel urgency might have different phenotypes that could affect their treatment outcomes, as bowel urgency improvement has been shown to be associated with better clinical outcomes and because bowel urgency can be independent of other symptom changes [15]. In the current analyses, a greater proportion of patients achieved clinical endpoints with mirikizumab versus placebo regardless of their Urgency NRS score (0–3, 4–6, or 7–10) at induction baseline, suggesting potential efficacy across phenotypes represented by various levels of baseline disease severity.

Following normalization of mucosal inflammation with induction therapy, continued long-term treatment with mirikizumab may allow additional improvement through two mechanisms. First, by way of molecular healing, mirikizumab upregulates colonic gene transcription that targets drivers of disease in patients with prior failure of biologic therapy, which is linked to mucosal healing and associated with decreased inflammation [33]. These transcriptional changes correlate with disease activity assessments and demonstrate a profile of disease attenuation associated with improved histologic outcomes. This mucosal healing appears to occur through gene products representing both structural components of healthy mucosa and crucial functional proteins, such as water and efflux transporters and adhesion molecules. Second, perhaps in connection with molecular healing, tissue repair mechanisms that are allowed to take place due to the amelioration of overactive inflammation, leading to physical changes in the gut, may move toward normalization over time [34].

The complexity of long-term improvement in bowel urgency and underlying healing is notable in these post hoc analyses for two reasons. First, when examining the week 52 data, it is essential to note that placebo-treated patients were responders to mirikizumab induction therapy who were rerandomized to placebo (mirikizumab treatment withdrawal) at week 12. Based on the 9.5-day half-life of mirikizumab [35] and the fact that these patients had received mirikizumab for 12 weeks, placebo-treated patients during the maintenance study may have maintained a level of efficacy due to a break in inflammatory responses that allowed some degree of system normalization. Second, since bowel urgency in patients with long-term disease may be multifactorial, including structural and neural changes that could take longer to heal than the underlying inflammation, bowel urgency improvements may not coincide with improvements in mucosal inflammation or track with other symptom improvement following treatment. In fact, patients can report bowel urgency even if they are considered in remission based on stool frequency, rectal bleeding, and endoscopy, and approximately 35%–40% of patients with no rectal bleeding or normal stool frequency still experience urgency [8]. These observations highlight the clinical relevance of bowel urgency and the importance of assessing improvement in severity over time.

While the clinical implications of bowel urgency improvement on long-term clinical outcomes have not been fully elucidated, data suggest that improvement in bowel urgency is associated with improved clinical outcomes [15]. Nevertheless, a maintained mean change in Urgency NRS score across patients over time may suggest that urgency could improve regardless of other patient outcomes. Responders to mirikizumab induction treatment who continued on to maintenance treatment maintained a mean change from baseline in the Urgency NRS score through week 52 (−3.80 ± 0.14) [15]. For patients who continued on extension treatment, the mean change from baseline in the Urgency NRS score was maintained through week 104 (−4.44 ± 0.11) [36]. Considering that bowel urgency matters to patients [17], treatment-induced change in bowel urgency and how it relates to outcomes is important. The current results help provide insights, showing that treatment with mirikizumab has a greater effect on change in bowel urgency than does placebo, independent of other outcomes and irrespective of the patient’s starting level of bowel urgency.

**Fig. 5:**
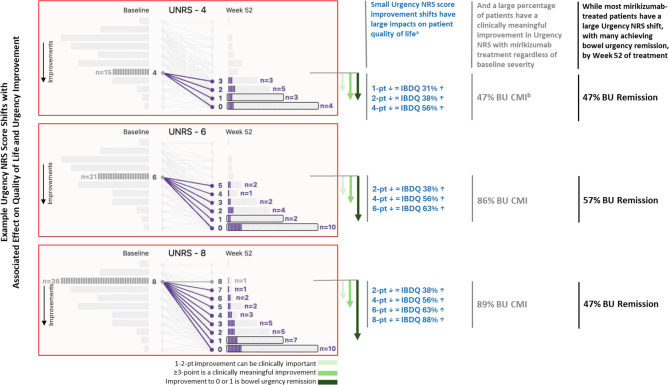
Clinical relevance of Urgency NRS score improvement shifts. ^a^IBDQ data shown are based on the evaluable overall population (n = 26, 39, 49, 32, and 25 for 1, 2, 4, 6, and 8-point Urgency NRS reductions, respectively), not the n from the individual shift examples shown, with the 8-pt data based upon 8 to 10-pt reductions. ^b^Patients with a low Urgency NRS at baseline have less of an opportunity to have a 3-point improvement

It has been shown that patients with UC prefer a bowel urgency scale that distinguishes levels of severity instead of categorical “yes/no” response options [37]. Ongoing assessment of bowel urgency severity can be important for tracking a patient’s clinical outcomes. Five factors support the clinical relevance of assessing the severity of bowel urgency over time (Supplementary Figure 5). First, a significantly greater proportion of patients who achieved clinically meaningful improvement or remission of bowel urgency, compared with those who did not, also achieved endpoints for symptomatic, clinical, and corticosteroid-free remission as well as clinical response, normalized fecal calprotectin and C-reactive protein, and improved health-related quality of life [15]. Second, patients have identified bowel urgency as one of the most bothersome symptoms that they want improved [8–10], which can take longer and track separately from other symptoms [15]. Third, when patients show bowel urgency improvement during mirikizumab therapy, they may improve anywhere from 1 to 10 points on the Urgency NRS, depending on their baseline starting point. A majority of patients have clinically meaningful improvement, with a large proportion reaching bowel urgency remission (Fig. 5). Mirikizumab responders tended to spread across the range of available improvement scores such that some only had 1-, 2-, or 3-point improvements. In cognitive debriefing interviews from a qualitative research study in adults with UC using the Urgency NRS, 9 of 16 patients reported that even a 1-point improvement was meaningful [17]. An additional 3 of 16 patients felt that a 2-point reduction was meaningful. Since a 1- to 2-point change in the Urgency NRS score can be important to patients, and a ≥ 3-point improvement has been shown to be clinically meaningful regardless of whether the patient is still above a score of 0 or 1 (urgency remission) [15–17], these changes are important and should be monitored. Fourth, small bowel urgency improvements (even one point) are associated with significant health-related quality of life improvements (Fig. 5). Each additional 1-point reduction in the Urgency NRS score corresponds to improvements in patient-reported outcomes based on the Inflammatory Bowel Disease Questionnaire. Fifth, patient response trajectory analyses have identified different mirikizumab patient response patterns called Super Responders, Responders, Delayed Responders, and Non/Incomplete Responders [38–40]. It was shown that a clinically meaningful improvement in Week 8 bowel urgency was a marker of being a mirikizumab Super Responder [38]. Helping patients understand that a small improvement could mean a lot in terms of changes to their health-related quality of life and ability to achieve long-term clinical outcomes, regardless of their baseline Urgency NRS score, may give those with bowel urgency more optimism about their health situation.

Study limitations to consider when interpreting these results include that these were post hoc analyses that were not controlled for multiplicity. The week 52 data are from the maintenance withdrawal LUCENT-2 study, where responders to mirikizumab induction treatment at week 12 were rerandomized to continue mirikizumab treatment or were withdrawn from mirikizumab treatment (placebo group). Thus, it is not a true placebo group as in the LUCENT-1 induction study comparisons, and patients in the mirikizumab arm had selection bias to those patients who responded to treatment in the induction study. Nonresponder imputation was only used to handle missing data, whereas patients who discontinued treatment or were missing endpoint assessments were treated as nonresponders. Nonresponder imputation is a conservative analytical approach and can be biased to show low remission/response rates.

## Conclusions

Regardless of baseline bowel urgency severity based on the Urgency NRS score, mirikizumab was efficacious in achieving symptomatic, endoscopic, and clinical endpoints in patients with moderately to severely active UC. Mirikizumab improved bowel urgency severity versus placebo, even among patients who did not achieve other clinical endpoints. Small bowel urgency improvements are associated with large health-related quality of life improvements. Thus, observing shifts in Urgency NRS scores along the Urgency NRS, regardless of the starting point, can aid in understanding a patient’s efficacy outcomes. The results of this study support the efficacy of mirikizumab in bowel urgency and the clinical relevance of assessing bowel urgency severity over time, as even small changes in severity can be important for patients.

## Electronic supplementary material

Below is the link to the electronic supplementary material.

Supplementary Material 1

Supplementary Material 2

## Data Availability

Eli Lilly and Company provides access to all individual participant data collected during the study, after anonymization. Data are available to request 6 months after the indication studied has been approved in the United States and the European Union and after primary publication acceptability, whichever is later. No expiration date for data requests is currently set once data are made available. Access is provided after a proposal has been approved by an independent review committee identified for this purpose and after receipt of a signed data sharing agreement. Data and documents, including the study protocol, statistical analysis plan, and study report, will be provided in a secure data sharing environment. For details on submitting a request, see the instructions provided at www.vivli.org.

## Abbreviations

BU: Bowel urgency
IBDQ: Inflammatory Bowel Disease Questionnaire
MIRI: Mirikizumab
NRI: Nonresponder imputation
NRS: Numeric Rating Scale
PBO: Placebo
UC: Ulcerative colitis
UNRS: Urgency Numeric Rating Scale
