# Supplementary material for: Bowel urgency in ulcerative colitis: effect of baseline urgency and change in urgency in response to mirikizumab
Source: J Patient Rep Outcomes. 2025 Jul 1;9:75. doi: 10.1186/s41687-025-00906-0 (PMC12214185; doi:10.1186/s41687-025-00906-0)
Supplement: Supplementary file 1 — Supplementary Material 1 [file 41687_2025_906_MOESM1_ESM.pdf]

SUPPLEMENTARY MATERIAL

Supplementary Table 1. Proportion of patients achieving efficacy endpoints at weeks 12 and 52 with mirikizumab versus placebo by Urgency NRS score at induction baseline.

	**Week 12**						**Week 52**					
	**Urgency NRS score**						**Urgency NRS score**					
	**0–3**		**4–6**		**7–10**		**0–3**		**4–6**		**7–10**	
**Efficacy endpoint**	**PBO (N = 37)**	**MIRI (N = 112)**	**PBO (N = 105)**	**MIRI (N = 332)**	**PBO (N = 152)**	**MIRI (N = 424)**	**PBO (N = 15)**	**MIRI (N = 55)**	**PBO (N = 77)**	**MIRI (N = 138)**	**PBO (N = 87)**	**MIRI (N = 172)**
Alternative clinical remission	4 (11)	40 (36)	18 (17)	91 (27)	21 (14)	91 (22)	3 (20)	29 (53)	23 (30)	66 (48)	21 (24)	94 (55)
BU CMI	0 (0)	7 (13)[b]	31 (30)	145 (44)	58 (38)	243 (57)	1 (13)	13 (50)[b]	33 (43)	81 (59)	38 (44)	125 (73)
BU remission	1 (5)	25 (46)[b]	21 (20)	85 (26)[a]	12 (8)	69 (16)	1 (13)	18 (69)[b]	22 (29)	61 (44)	20 (23)	65 (38)
Clinical remission	3 (8)	34 (30)	17 (16)	85 (26)	19 (13)	91 (22)	3 (20)	25 (46)[a]	21 (27)	65 (47)	21 (24)	92 (54)
Clinical response	10 (27)	71 (63)	45 (43)	219 (66)	69 (45)	261 (62)	5 (33)	43 (78)	43 (56)	112 (81)	40 (46)	138 (80)
CSF remission	NA	NA	NA	NA	NA	NA	2 (13)	21 (38)[a]	18 (23)	59 (43)	19 (22)	84 (49)
Endoscopic remission	4 (11)	50 (45)	24 (23)	126 (38)	34 (22)	139 (33)	3 (20)	32 (58)	25 (33)	74 (54)	24 (28)	108 (63)
Endoscopic response	10 (27)	68 (61)	40 (38)	181 (55)	56 (37)	232 (55)	6 (40)	38 (69)[a]	33 (43)	95 (69)	34 (39)	132 (77)
HEMR	0 (0)	32 (29)	13 (12)	90 (27)	20 (13)	71 (17)[a]	3 (20)	25 (46)[a]	18 (23)	56 (41)	18 (21)	77 (45)
IBDQ remission	22 (60)	86 (77)[a]	44 (42)	214 (65)	51 (34)	199 (47)	7 (47)	43 (78)	36 (47)	106 (77)	34 (39)	115 (67)
RB remission	18 (49)	73 (65)[a]	50 (48)	227 (68)	61 (40)	255 (60)	5 (33)	44 (80)	44 (57)	114 (83)	40 (46)	133 (77)
SF remission	11 (30)	67 (60)	47 (45)	208 (63)	59 (39)	220 (52)	6 (40)	38 (69)[a]	38 (49)	112 (81)	36 (41)	124 (72)
Symptomatic remission	8 (22)	52 (46)	35 (33)	169 (51)	39 (26)	174 (41)	4 (27)	37 (67)	35 (46)	106 (77)	32 (37)	116 (67)
Symptomatic response	16 (43)	76 (68)	57 (54)	249 (75)	81 (53)	300 (71)	7 (47)	45 (82)	47 (61)	117 (85)	44 (51)	142 (83)

Data are shown as n (%) of modified intention-to-treat patients meeting the specified endpoint at week 12 and week 52. Week 12: PBO, N = 294; MIRI, N = 868. Week 52: PBO, N = 179; MIRI, N = 365. The efficacy endpoints of BU CMI and BU remission were only calculated for patients with an Urgency NRS score ≥3 at baseline, so the numbers of patients in the Urgency NRS 0–3 score group for PBO and MIRI, respectively, are 19 and 55 at week 12 and 8 and 26 at week 52. Percentages are rounded to the nearest whole number, with 0.5 rounded up and 0.4 rounded down. See *Efficacy Endpoints* section for definitions. Using logistic regression and Fisher's exact test, all MIRI versus PBO treatment comparisons are statistically significant at $P < 0.05$ unless marked [a] or [b].

[a]Not statistically significant at $P < 0.05$.

[b]Could not be assessed due to low n.

Abbreviations: BU, bowel urgency; CI, confidence interval; CMI, clinically meaningful improvement; CSF, corticosteroid-free remission; HEMR, histologic-endoscopic mucosal remission; IBDQ, Inflammatory Bowel Disease Questionnaire; MIRI, mirikizumab; *N*, number of patients in the analysis population OR the number of patients in each Urgency NRS score grouping; *n*, number of patients within analyzed group achieving the endpoint of interest; NA, not applicable; NRS, Numeric Rating Scale; PBO, placebo; RB, rectal bleeding; SF, stool frequency.

Supplementary Table 2. Shift from LUCENT-1 induction baseline to week 12 or 52 by Urgency NRS score group for mirikizumab- and placebo-treated patients achieving or not achieving (A) BU CMI; (B) BU remission; (C) clinical remission; (D) clinical response; (E) CSF remission; (F) endoscopic remission; (G) HEMR; (H) IBDQ remission; and (I) symptomatic remission.

**(A)**<br>**BU CMI[a]**<br>**(*N* at week 12/52)**	**Baseline Urgency NRS score groups[b] (*N* at week 12/52)**	**Week 12 Urgency NRS score groups,[b,c] *n* (%)**<br>**0–3**<br>**[BU remission][e]**	**4–6**	**7–10**	**Week 52 Urgency NRS score groups,[b,c,d] *n* (%)**<br>**0–3**<br>**[BU remission][e]**	**4–6**	**7–10**
Mirikizumab, endpoint achieved (*N* = 395/219)	0–3 (*N* = 7/13)	7 (100)<br>[7 (100)]	0 (0.0)	0 (0.0)	13 (100)<br>[13 (100)]	0 (0.0)	0 (0.0)
	4–6 (*N* = 145/81)	145 (100)<br>[85 (58.6)]	0 (0.0)	0 (0.0)	81 (100)<br>[61 (75.3)]	0 (0.0)	0 (0.0)
	7–10 (*N* = 243/125)	166 (68.3)<br>[69 (28.4)]	75 (30.9)	2 (0.8)	108 (86.4)<br>[65 (52.0)]	16 (12.8)	1 (0.8)
Mirikizumab, endpoint not achieved (*N* = 416/117)	0–3 (*N* = 48/13)	38 (79.2)<br>[18 (37.5)]	9 (18.8)	1 (2.1)	10 (76.9)<br>[7 (53.8)]	3 (23.1)	0 (0.0)
	4–6 (*N* = 187/57)	63 (33.7)<br>[0 (0.0)]	103 (55.1)	21 (11.2)	32 (56.1)<br>[3 (5.3)]	22 (38.6)	3 (5.3)
	7–10 (*N* = 181/47)	2 (1.1)<br>[1 (0.6)]	60 (33.1)	119 (65.7)	8 (17.0)<br>[0 (0.0)]	12 (25.5)	27 (57.4)
Placebo, endpoint achieved (*N* = 89/72)	0–3 (*N* = 0/1)	0 (0.0)<br>[0 (0.0)]	0 (0.0)	0 (0.0)	1 (100)<br>[1 (100)]	0 (0.0)	0 (0.0)
	4–6 (*N* = 31/33)	31 (100)<br>[21 (67.7)]	0 (0.0)	0 (0.0)	33 (100)<br>[22 (66.7)]	0 (0.0)	0 (0.0)
	7–10 (*N* = 58/38)	35 (60.3)<br>[[12 (20.7)]	23 (39.7)	0 (0.0)	32 (84.2)<br>[20 (52.6)]	6 (15.8)	0 (0.0)
Placebo, endpoint not achieved (*N* = 187/100)	0–3 (*N* = 19/7)	13 (68.4)<br>[1 (5.3)]	5 (26.3)	1 (5.3)	4 (57.1)<br>[0 (0.0)]	3 (42.9)	0 (0.0)
	4–6 (*N* = 74/44)	17 (23.0)<br>[2 (2.7)]	49 (66.2)	8 (10.8)	12 (27.3)<br>[2 (4.5)]	24 (54.5)	8 (18.2)
	7–10 (*N* = 94/49)	0 (0.0)<br>[0 (0.0)]	27 (28.7)	67 (71.3)	5 (10.2)<br>[2 (4.1)]	16 (32.7)	28 (57.1)

(B)<br>BU remission[a]<br>(*N* at week 12/52)	Baseline Urgency NRS score groups[b] (*N* at week 12/52)	Week 12 Urgency NRS score groups,[b,c] *n* (%)			Week 52 Urgency NRS score groups,[b,c,d] *n* (%)		
		**0–3 [BU remission][e]**	**4–6**	**7–10**	**0–3 [BU remission][e]**	**4–6**	**7–10**
Mirikizumab, endpoint achieved (*N* = 179/144)	0–3 (*N* = 25/18)	25 (100)<br>[25 (100)]	0 (0.0)	0 (0.0)	18 (100)<br>[18 (100)]	0 (0.0)	0 (0.0)
	4–6 (*N* = 85/61)	85 (100)<br>[85 (100)]	0 (0.0)	0 (0.0)	61 (100)<br>[61 (100)]	0 (0.0)	0 (0.0)
	7–10 (*N* = 69/65)	69 (100)<br>[69 (100)]	0 (0.0)	0 (0.0)	65 (100)<br>[65 (100)]	0 (0.0)	0 (0.0)
Mirikizumab, endpoint not achieved (*N* = 632/192)	0–3 (*N* = 30/8)	20 (66.7)<br>[0 (0.0)]	9 (30.0)	1 (3.3)	5 (62.5)<br>[2 (25.0)]	3 (37.5)	0 (0.0)
	4–6 (*N* = 247/77)	123 (49.8)<br>0 (0.0)]	103 (41.7)	21 (8.5)	52 (67.5)<br>3 (3.9)]	22 (28.6)	3 (3.9)
	7–10 (*N* = 355/107)	99 (27.9)<br>[1 (0.3)]]	135 (38.0)	121 (34.1)	51 (47.7)<br>[0 (0.0)]	28 (26.2)	28 (26.2)
Placebo, endpoint achieved (*N* = 34/43)	0–3 (*N* = 1/1)	1 (100)<br>[1 (100)]	0 (0.0)	0 (0.0)	1 (100)<br>[1 (100)]	0 (0.0)	0 (0.0)
	4–6 (*N* = 21/22)	21 (100)<br>[21(100)]	0 (0.0)	0 (0.0)	22 (100)<br>[22 (100)]	0 (0.0)	0 (0.0)
	7–10 (*N* = 12/20)	12 (100)<br>[12 (100)]	0 (0.0)	0 (0.0)	20 (100)<br>[20 (100)]	0 (0.0)	0 (0.0)
Placebo, endpoint not achieved (*N* = 242/129)	0–3 (*N* = 18/7)	12 (66.7)<br>[0 (0.0)]	5 (27.8)	1 (5.6)	4 (57.1)<br>[0 (0.0)]	3 (42.9)	0 (0.0)
	4–6 (*N* = 84/55)	27 (32.1)<br>[2 (2.4)]	49 (58.3)	8 (9.5)	23 (41.8)<br>[2 (3.6)]	24 (43.6)	8 (14.5)
	7–10 (*N* = 140/67)	23 (16.4)<br>[0 (0.0)]	50 (35.7)	67 (47.9)	17 (25.4)<br>[2 (3.0)]	22 (32.8)	28 (41.8)

**(C)**<br>**Clinical remission[a]**<br>**(*N* at week 12/52)**	**Baseline Urgency NRS score groups[b] (*N* at week 12/52)**	**Week 12 Urgency NRS score groups,[b,c] *n* (%)**			**Week 52 Urgency NRS score groups,[b,c,d] *n* (%)**		
		**0–3 [BU remission][e]**	**4–6**	**7–10**	**0–3 [BU remission][e]**	**4–6**	**7–10**
Mirikizumab, endpoint achieved (*N* = 210/182)	0–3 (*N* = 34/25)	31 (91.2) [23 (67.6)]	3 (8.8)	0 (0.0)	23 (92.0) [18 (72.0)]	2 (8.0)	0 (0.0)
	4–6 (*N* = 85/65)	71 (83.5) [36 (42.4)]	12 (14.1)	2 (2.4)	58 (89.2) [36 (55.4)]	7 (10.8)	0 (0.0)
	7–10 (*N* = 91/92)	66 (72.5) [29 (31.9)]	19 (20.9)	6 (6.6)	73 (79.3) [49 (53.3)]	12 (13.0)	7 (7.6)
Mirikizumab, endpoint not achieved (*N* = 658/183)	0–3 (*N* = 78/30)	67 (85.9) [34 (43.6)]	8 (10.3)	3 (3.8)	28 (93.3) [20 (66.7)]	2 (6.7)	0 (0.0)
	4–6 (*N* = 247/73)	137 (55.5) [49 (19.8)]	91 (36.8)	19 (7.7)	55 (75.3) [28 (38.4)]	15 (20.5)	3 (4.1)
	7–10 (*N* = 333/80)	102 (30.6) [41 (12.3)]	116 (34.8)	115 (34.5)	43 (53.8) [16 (20.0)]	16 (20.0)	21 (26.3)
Placebo, endpoint achieved (*N* = 39/45)	0–3 (*N* = 3/3)	3 (100) [2 (66.7)]	0 (0.0)	0 (0.0)	3 (100) [2 (66.7)]	0 (0.0)	0 (0.0)
	4–6 (*N* = 17/21)	13 (76.5) [10 (58.8)]	4 (23.5)	0 (0.0)	19 (90.5) [15 (71.4)]	1 (4.8)	1 (4.8)
	7–10 (*N* = 19/21)	11 (57.9) [5 (26.3)]	6 (31.6)	2 (10.5)	20 (95.2) [13 (61.9)]	1 (4.8)	0 (0.0)
Placebo, endpoint not achieved (*N* = 255/134)	0–3 (*N* = 34/12)	24 (70.6) [8 (23.5)]	9 (26.5)	1 (2.9)	6 (50.0) [2 (16.7)]	4 (33.3)	2 (16.7)
	4–6 (*N* = 88/56)	35 (39.8) [13 (14.8)]	45 (51.1)	8 (9.1)	26 (46.4) [9 (16.1)]	23 (41.1)	7 (12.5)
	7–10 (*N* = 133/66)	24 (18.0) [7 (5.3)]	44 (33.1)	65 (48.9)	17 (25.8) [9 (13.6)]	21 (31.8)	28 (42.4)

(D)<br>Clinical response[a]<br>(*N* at week 12/52)	Baseline Urgency NRS score groups[b] (*N* at week 12/52)	Week 12 Urgency NRS score groups,[b,c] *n* (%)			Week 52 Urgency NRS score groups,[b,c,d] *n* (%)		
		0–3<br>[BU remission][e]	4–6	7–10	0–3<br>[BU remission][e]	4–6	7–10
Mirikizumab, endpoint achieved (*N* = 551/293)	0–3 (*N* = 71/43)	67 (94.4)<br>[46 (64.8)]	4 (5.6)	0 (0.0)	41 (95.3)<br>[31 (72.1)]	2 (4.7)	0 (0.0)
	4–6 (*N* = 219/112)	166 (75.8)<br>[72 (32.9)]	47 (21.5)	6 (2.7)	101 (90.2)<br>[57 (50.9)]	10 (8.9)	1 (0.9)
	7–10 (*N* = 261/138)	148 (56.7)<br>[67 (25.7)]	80 (30.7)	33 (12.6)	105 (76.1)<br>[64 (46.4)]	21 (15.2)	12 (8.7)
Mirikizumab, endpoint not achieved (*N* = 317/72)	0–3 (*N* = 41/12)	31 (75.6)<br>[11 (26.8)]	7 (17.1)	3 (7.3)	10 (83.3)<br>[7 (58.3)]	2 (16.7)	0 (0.0)
	4–6 (*N* = 113/26)	42 (37.2)<br>[13 (11.5)]	56 (49.6)	15 (13.3)	12 (46.2)<br>[7 (26.9)]	12 (46.2)	2 (7.7)
	7–10 (*N* = 163/34)	20 (12.3)<br>[3 (1.8)]	55 (33.7)	88 (54.0)	11 (32.4)<br>[1 (2.9)]	7 (20.6)	16 (47.1)
Placebo, endpoint achieved (*N* = 124/88)	0–3 (*N* = 10/5)	8 (80.0)<br>[4 (40)]	2 (20.0)	0 (0.0)	5 (100)<br>[3 (60.0)]	0 (0.0)	0 (0.0)
	4–6 (*N* = 45/43)	35 (77.8)<br>[21 (46.7)]	9 (20.0)	1 (2.2)	36 (83.7)<br>[21 (48.8)]	5 (11.6)	2 (4.7)
	7–10 (*N* = 69/40)	31 (44.9)<br>[11 (15.9)]	28 (40.6)	10 (14.5)	32 (80.0)<br>[19 (47.5)]	7 (17.5)	1 (2.5)
Placebo, endpoint not achieved (*N* = 170/91)	0–3 (*N* = 27/10)	19 (70.4)<br>[6 (22.2)]	7 (25.9)	1 (3.7)	4 (40.0)<br>[1 (10.0)]	4 (40.0)	2 (20.0)
	4–6 (*N* = 60/34)	13 (21.7)<br>[2 (3.3)]	40 (66.7)	7 (11.7)	9 (26.5)<br>[3 (8.8)]	19 (55.9)	6 (17.6)
	7–10 (*N* = 83/47)	4 (4.8)<br>[1 (1.2)]	22 (26.5)	57 (68.7)	5 (10.6)<br>[3 (6.4)]	15 (31.9)	27 (57.4)

(E) CSF remission[a] (*N* at week 12/52)	Baseline Urgency NRS score groups[b] (*N* at week 12/52)	Week 12 Urgency NRS score groups,[b,c] *n* (%)			Week 52 Urgency NRS score groups,[b,c,d] *n* (%)		
		0–3 [BU remission][e]	4–6	7–10	0–3 [BU remission][e]	4–6	7–10
Mirikizumab, endpoint achieved (*N* = NA/164)	0–3 (*N* = NA/21)	NA	NA	NA	19 (90.5) [15 (71.4)]	2 (9.5)	0 (0.0)
	4–6 (*N* = NA/59)	NA	NA	NA	52 (88.1) [32 (54.2)]	7 (11.9)	0 (0.0)
	7–10 (*N* = NA/84)	NA	NA	NA	69 (82.1) [48 (57.1)]	10 (11.9)	5 (6.0)
Mirikizumab, endpoint not achieved (*N* = NA/201)	0–3 (*N* = NA/34)	NA	NA	NA	32 (94.1) [23 (67.6)]	2 (5.9)	0 (0.0)
	4–6 (*N* = NA/79)	NA	NA	NA	61 (77.2) [32 (40.5)]	15 (19.0)	3 (3.8)
	7–10 (*N* = NA/88)	NA	NA	NA	47 (53.4) [17 (19.3)]	18 (20.5)	23 (26.1)
Placebo, endpoint achieved (*N* = NA/39)	0–3 (*N* = NA/2)	NA	NA	NA	2 (100) [2 (100)]	0 (0.0)	0 (0.0)
	4–6 (*N* = NA/18)	NA	NA	NA	16 (88.9) [12 (66.7)]	1 (5.6)	1 (5.6)
	7–10 (*N* = NA/19)	NA	NA	NA	18 (94.7) [12 (63.2)]	1 (5.3)	0 (0.0)
Placebo, endpoint not achieved (*N* = NA/140)	0–3 (*N* = NA/13)	NA	NA	NA	7 (53.8) [2 (15.4)]	4 (30.8)	2 (15.4)
	4–6 (*N* = NA/59)	NA	NA	NA	29 (49.2) [12 (20.3)]	23 (39.0)	7 (11.9)
	7–10 (*N* = NA/68)	NA	NA	NA	19 (27.9) [10 (14.7)]	21 (30.9)	28 (41.2)

(F) Endoscopic remission[a] (N at week 12/52)	Baseline Urgency NRS score groups[b] (N at week 12/52)	Week 12 Urgency NRS score groups,[b,c] n (%)			Week 52 Urgency NRS score groups,[b,c,d] n (%)		
		0–3 [BU remission][e]	4–6	7–10	0–3 [BU remission][e]	4–6	7–10
Mirikizumab, endpoint achieved ($N = 315/214$)	0–3 ($N = 50/32$)	47 (94.0) [32 (64.0)]	3 (6.0)	0 (0.0)	29 (90.6) [22 (68.8)]	3 (9.4)	0 (0.0)
	4–6 ($N = 126/74$)	97 (77.0) [41 (32.5)]	24 (19.0)	5 (4.0)	66 (89.2) [39 (52.7)]	8 (10.8)	0 (0.0)
	7–10 ($N = 139/108$)	84 (60.4) [37 (26.6)]	36 (25.9)	19 (13.7)	82 (75.9) [51 (47.2)]	16 (14.8)	10 (9.3)
Mirikizumab, endpoint not achieved ($N = 553/151$)	0–3 ($N = 62/23$)	51 (82.3) [25 (40.3)]	8 (12.9)	3 (4.8)	22 (95.7) [16 (69.6)]	1 (4.3)	0 (0.0)
	4–6 ($N = 206/64$)	111 (53.9) [44 (21.4)]	79 (38.3)	16 (7.8)	47 (73.4) [25 (39.1)]	14 (21.9)	3 (4.7)
	7–10 ($N = 285/64$)	84 (29.5) [33 (11.6)]	99 (34.7)	102 (35.8)	34 (53.1) [14 (21.9)]	12 (18.8)	18 (28.1)
Placebo, endpoint achieved ($N = 62/52$)	0–3 ($N = 4/3$)	4 (100) [2 (50.0)]	0 (0.0)	0 (0.0)	3 (100) [2 (66.7)]	0 (0.0)	0 (0.0)
	4–6 ($N = 24/25$)	19 (79.2) [11 (45.8)]	5 (20.8)	0 (0.0)	21 (84.0) [15 (60.0)]	2 (8.0)	2 (8.0)
	7–10 ($N = 34/24$)	14 (41.2) [6 (17.6)]	15 (44.1)	5 (14.7)	21 (87.5) [14 (58.3)]	3 (12.5)	0 (0.0)
Placebo, endpoint not achieved ($N = 232/127$)	0–3 ($N = 33/12$)	23 (69.7) [8 (24.2)]	9 (27.3)	1 (3.0)	6 (50.0) [2 (16.7)]	4 (33.3)	2 (16.7)
	4–6 ($N = 81/52$)	29 (35.8) [12 (14.8)]	44 (54.3)	8 (9.9)	24 (46.2) [9 (17.3)]	22 (42.3)	6 (11.5)
	7–10 ($N = 118/63$)	21 (17.8) [6 (5.1)]	35 (29.7)	62 (52.5)	16 (25.4) [8 (12.7)]	19 (30.2)	28 (44.4)

**(G)**		**Week 12 Urgency NRS score groups,[b,c] *n* (%)**			**Week 52 Urgency NRS score groups,[b,c,d] *n* (%)**		
**HEMR[a] (*N* at week 12/52)**	**Baseline Urgency NRS score groups[b] (*N* at week 12/52)**	**0–3 [BU remission][e]**	**4–6**	**7–10**	**0–3 [BU remission][e]**	**4–6**	**7–10**
Mirikizumab, endpoint achieved (*N* = 193/158)	0–3 (*N* = 32/25)	31 (96.9) [22 (68.8)]	1 (3.1)	0 (0.0)	23 (92.0) [17 (68.0)]	2 (8.0)	0 (0.0)
	4–6 (*N* = 90/56)	72 (80.0) [29 (32.2)]	15 (16.7)	3 (3.3)	50 (89.3) [26 (46.4)]	6 (10.7)	0 (0.0)
	7–10 (*N* = 71/77)	38 (53.5) [18 (25.4)]	21 (29.6)	12 (16.9)	57 (74.0) [37 (48.1)]	11 (14.3)	9 (11.7)
Mirikizumab, endpoint not achieved (*N* = 675/207)	0–3 (*N* = 80/30)	67 (83.8) [35 (43.8)]	10 (12.5)	3 (3.8)	28 (93.3) [21 (70.0)]	2 (6.7)	0 (0.0)
	4–6 (*N* = 242/82)	136 (56.2) [56 (23.1)]	88 (36.4)	18 (7.4)	63 (76.8) [38 (46.3)]	16 (19.5)	3 (3.7)
	7–10 (*N* = 353/95)	130 (36.8) [52 (14.7)]	114 (32.3)	109 (30.9)	59 (62.1) [28 (29.5)]	17 (17.9)	19 (20.0)
Placebo, endpoint achieved (*N* = 33/39)	0–3 (*N* = 0/3)	0 (0.0) [0 (0.0)]	0 (0.0)	0 (0.0)	3 (100) [2 (66.7)]	0 (0.0)	0 (0.0)
	4–6 (*N* = 13/18)	11 (84.6) [6 (46.2)]	2 (15.4)	0 (0.0)	15 (83.3) [9 (50.0)]	1 (5.6)	2 (11.1)
	7–10 (*N* = 20/18)	11 (55.0) [5 (25.0)]	8 (40.0)	1 (5.0)	17 (94.4) [12 (66.7)]	1 (5.6)	0 (0.0)
Placebo, endpoint not achieved (*N* = 261/140)	0–3 (*N* = 37/12)	27 (73.0) [10 (27.0)]	9 (24.3)	1 (2.7)	6 (50.0) [2 (16.7)]	4 (33.3)	2 (16.7)
	4–6 (*N* = 92/59)	37 (40.2) [17 (18.5)]	47 (51.1)	8 (8.7)	30 (50.8) [15 (25.4)]	23 (39.0)	6 (10.2)
	7–10 (*N* = 132/69)	24 (18.2) [7 (5.3)]	42 (31.8)	66 (50.0)	20 (29.0) [10 (14.5)]	21 (30.4)	28 (40.6)

(H)<br>IBDQ[a]<br>(*N* at week 12/52)	Baseline Urgency NRS score groups[b] (*N* at week 12/52)	Week 12 Urgency NRS score groups,[b,c] *n* (%)			Week 52 Urgency NRS score groups,[b,c,d] *n* (%)		
		0–3<br>[BU remission][e]	4–6	7–10	0–3<br>[BU remission][e]	4–6	7–10
Mirikizumab, endpoint achieved (*N* = 499/264)	0–3 (*N* = 86/43)	80 (93.0)<br>[52 (60.5)]	6 (7.0)	0 (0.0)	40 (93.0)<br>[32 (74.4)]	3 (7.0)	0 (0.0)
	4–6 (*N* = 214/106)	165 (77.1)<br>[76 (35.5)]	44 (20.6)	5 (2.3)	95 (89.6)<br>[56 (52.8)]	10 (9.4)	1 (0.9)
	7–10 (*N* = 199/115)	117 (58.8)<br>[60 (30.2)]	51 (25.6)	31 (15.6)	91 (79.1)<br>[59 (51.3)]	17 (14.8)	7 (6.1)
Mirikizumab, endpoint not achieved (*N* = 369/101)	0–3 (*N* = 26/12)	18 (69.2)<br>[5 (19.2)]	5 (19.2)	3 (11.5)	11 (91.7)<br>[6 (50.0)]	1 (8.3)	0 (0.0)
	4–6 (*N* = 118/32)	43 (36.4)<br>[9 (7.6)]	59 (50.0)	16 (13.6)	18 (56.3)<br>[8 (25.0)]	12 (37.5)	2 (6.3)
	7–10 (*N* = 225/57)	51 (22.7)<br>[10 (4.4)]	84 (37.3)	90 (40.0)	25 (43.9)<br>[6 (10.5)]	11 (19.3)	21 (36.8)
Placebo, endpoint achieved (*N* = 117/77)	0–3 (*N* = 22/7)	18 (81.8)<br>[9 (41.0)]	4 (18.2)	0 (0.0)	7 (100)<br>[4 (57.1)]	0 (0.0)	0 (0.0)
	4–6 (*N* = 44/36)	28 (63.6)<br>[17 (38.6)]	16 (36.4)	0 (0.0)	32 (88.9)<br>[20 (55.6)]	4 (11.1)	0 (0.0)
	7–10 (*N* = 51/34)	22 (43.1)<br>[9 (17.6)]	18 (35.3)	11 (21.6)	30 (88.2)<br>[20 (58.8)]	4 (11.8)	0 (0.0)
Placebo, endpoint not achieved (*N* = 177/102)	0–3 (*N* = 15/8)	9 (60.0)<br>[1 (6.7)]	5 (33.3)	1 (6.7)	2 (25.0)<br>[0 (0.0)]	4 (50.0)	2 (25.0)
	4–6 (*N* = 61/41)	20 (32.8)<br>[6 (9.8)]	33 (54.1)	8 (13.1)	13 (31.7)<br>[4 (9.8)]	20 (48.8)	8 (19.5)
	7–10 (*N* = 101/53)	13 (12.9)<br>[3 (3.0)]	32 (31.7)	56 (55.4)	7 (13.2)<br>[2 (3.8)]	18 (34.0)	28 (52.8)

**(I) Symptomatic remission[a] (*N* at week 12/52)**	**Baseline Urgency NRS score groups[b] (*N* at week 12/52)**	**Week 12 Urgency NRS score groups,[b,c] *n* (%)**			**Week 52 Urgency NRS score groups,[b,c,d] *n* (%)**		
		**0–3 [BU remission][e]**	**4–6**	**7–10**	**0–3 [BU remission][e]**	**4–6**	**7–10**
Mirikizumab, endpoint achieved (*N* = 395/259)	0–3 (*N* = 52/37)	49 (94.2) [35 (67.3)]	3 (5.8)	0 (0.0)	35 (94.6) [27 (73.0)]	2 (5.4)	0 (0.0)
	4–6 (*N* = 169/106)	133 (78.7) [64 (37.9)]	32 (18.9)	4 (2.4)	95 (89.6) [57 (53.8)]	10 (9.4)	1 (0.9)
	7–10 (*N* = 174/116)	119 (68.4) [56 (32.2)]	44 (25.3)	11 (6.3)	94 (81.0) [62 (53.4)]	15 (12.9)	7 (6.0)
Mirikizumab, endpoint not achieved (*N* = 473/106)	0–3 (*N* = 60/18)	49 (81.7) [22 (36.7)]	8 (13.3)	3 (5.0)	16 (88.9) [11 (61.1)]	2 (11.1)	0 (0.0)
	4–6 (*N* = 163/32)	75 (46.0) [21 (12.9)]	71 (43.6)	17 (10.4)	18 (56.3) [7 (21.9)]	12 (37.5)	2 (6.3)
	7–10 (*N* = 250/56)	49 (19.6) [14 (5.6)]	91 (36.4)	110 (44.0)	22 (39.3) [3 (5.4)]	13 (23.2)	21 (37.5)
Placebo, endpoint achieved (*N* = 82/71)	0–3 (*N* = 8/4)	7 (87.5) [4 (50.0)]	1 (12.5)	0 (0.0)	4 (100) [2 (50.0)]	0 (0.0)	0 (0.0)
	4–6 (*N* = 35/35)	26 (74.3) [18 (51.4)]	8 (22.9)	1 (2.9)	31 (88.6) [20 (57.1)]	3 (8.6)	1 (2.9)
	7–10 (*N* = 39/32)	23 (59.0) [10 (25.6)]	11 (28.2)	5 (12.8)	29 (90.6) [17 (53.1)]	2 (6.3)	1 (3.1)
Placebo, endpoint not achieved (*N* = 212/108)	0–3 (*N* = 29/11)	20 (69.0) [6 (20.7)]	8 (27.6)	1 (3.4)	5 (45.5) [2 (18.2)]	4 (36.4)	2 (18.2)
	4–6 (*N* = 70/42)	22 (31.4) [5 (7.1)]	41 (58.6)	7 (10.0)	14 (33.3) [4 (9.5)]	21 (50.0)	7 (16.7)
	7–10 (*N* = 113/55)	12 (10.6) [2 (1.8)]	39 (34.5)	62 (54.9)	8 (14.5) [5 (9.1)]	20 (36.4)	27 (49.1)

N = X/X refers to patient numbers for that line item at week 12 and week 52, respectively. Shaded cells indicate the following: medium gray = improved ≥1 point; dark gray = unchanged; and light gray = worsened ≥1 point.

[a]See the *Efficacy Endpoints* section for definitions.

[b]Urgency NRS score groups included 0–3 (BU remission: 0–1), 4–6, and 7–10.

[c]Missing responses were imputed using nonresponder imputation.

[d]Patients at week 52 were responders to mirikizumab induction therapy at week 12 who were rerandomized to mirikizumab or placebo.

[e]Urgency NRS score of 0 or 1 refers to BU remission and is a subcategory of the Urgency NRS 0–3 score group.

Abbreviations: BU, bowel urgency; CMI, clinically meaningful improvement; CSF, corticosteroid-free remission; HEMR, histologic-endoscopic mucosal remission; IBDQ, Inflammatory Bowel Disease Questionnaire; *N*, number of patients in the analysis population; *n*, number of patients within analyzed group achieving the endpoint of interest; NA, not applicable; NRS, Numeric Rating Scale.

Supplementary Table 3. Group score shifts in Urgency NRS score from induction baseline to weeks 12 and 52 for mirikizumab- and placebo-treated patients who did and did not achieve clinical remission.

Clinical remission (*N* at week 12/52)	Baseline Urgency NRS score groups[a] (*N* at week 12/52)	Week 12 Urgency NRS score group,[b] *n* (%)			Week 52 Urgency NRS score group,[b,c] *n* (%)		
		0–3	4–6	7–10	0–3	4–6	7–10
Mirikizumab remitter (*N* = 210/182)	0–3 (*N* = 34/25)	31 (91.2)	3 (8.8)	0 (0.0)	23 (92.0)	2 (8.0)	0 (0.0)
	4–6 (*N* = 85/65)	71 (83.5)	12 (14.1)	2 (2.4)	58 (89.2)	7 (10.8)	0 (0.0)
	7–10 (*N* = 91/92)	66 (72.5)	19 (20.9)	6 (6.6)	73 (79.3)	12 (13.0)	7 (7.6)
Mirikizumab nonremitter (*N* = 658/183)	0–3 (*N* = 78/30)	67 (85.9)	8 (10.3)	3 (3.8)	28 (93.3)	2 (6.7)	0 (0.0)
	4–6 (*N* = 247/73)	137 (55.5)	91 (36.8)	19 (7.7)	55 (75.3)	15 (20.5)	3 (4.1)
	7–10 (*N* = 333/80)	102 (30.6)	116 (34.8)	115 (34.5)	43 (53.8)	16 (20.0)	21 (26.3)
Placebo remitter (*N* = 39/45)	0–3 (*N* = 3/3)	3 (100)	0 (0.0)	0 (0.0)	3 (100)	0 (0.0)	0 (0.0)
	4–6 (*N* = 17/21)	13 (76.5)	4 (23.5)	0 (0.0)	19 (90.5)	1 (4.8)	1 (4.8)
	7–10 (*N* = 19/21)	11 (57.9)	6 (31.6)	2 (10.5)	20 (95.2)	1 (4.8)	0 (0.0)
Placebo nonremitter (*N* = 255/134)	0–3 (*N* = 34/12)	24 (70.6)	9 (26.5)	1 (2.9)	6 (50.0)	4 (33.3)	2 (16.7)
	4–6 (*N* = 88/56)	35 (39.8)	45 (51.1)	8 (9.1)	26 (46.4)	23 (41.1)	7 (12.5)
	7–10 (*N* = 133/66)	24 (18.0)	44 (33.1)	65 (48.9)	17 (25.8)	21 (31.8)	28 (42.4)

N = X/X refers to patient numbers for that line item at week 12 and week 52, respectively. Shaded cells indicate the following: Purple = improved; gray = unchanged; orange = worsened.

[a]Urgency NRS score groups included 0–3, 4–6, and 7–10.

[b]Missing responses were imputed using nonresponder imputation.

[c]Patients at week 52 were responders to mirikizumab induction therapy at week 12 who were rerandomized to mirikizumab or placebo.

Abbreviation: *N*, number of patients in the analysis population; *n*, number of patients within analyzed group achieving the endpoint of interest; NRS, Numeric Rating Scale.

Supplementary Table 4. Individual-point shift in Urgency NRS score from induction baseline to week 52 for (A) mirikizumab- and (B) placebo-treated patients who did and did not achieve clinical remission.

**(A) Mirikizumab-treated patients**											
**Baseline Urgency NRS score**	**Week 52 Urgency NRS score, *n* (%)**										
	**0**	**1**	**2**	**3**	**4**	**5**	**6**	**7**	**8**	**9**	**10**
**Patients that achieved clinical remission (*N* = 182) – UNRS score shift from induction baseline to week 52 for mirikizumab treated patients**											
0	1 (50.0)	1 (50.0)	0 (0.0)	0 (0.0)	0 (0.0)	0 (0.0)	0 (0.0)	0 (0.0)	0 (0.0)	0 (0.0)	0 (0.0)
1	2 (66.7)	0 (0.0)	1 (33.3)	0 (0.0)	0 (0.0)	0 (0.0)	0 (0.0)	0 (0.0)	0 (0.0)	0 (0.0)	0 (0.0)
2	1 (20.0)	1 (20.0)	2 (40.0)	1 (20.0)	0 (0.0)	0 (0.0)	0 (0.0)	0 (0.0)	0 (0.0)	0 (0.0)	0 (0.0)
3	8 (53.3)	4 (26.7)	1 (6.7)	0 (0.0)	1 (6.7)	1 (6.7)	0 (0.0)	0 (0.0)	0 (0.0)	0 (0.0)	0 (0.0)
4	4 (26.7)	3 (20.0)	5 (33.3)	3 (20.0)	0 (0.0)	0 (0.0)	0 (0.0)	0 (0.0)	0 (0.0)	0 (0.0)	0 (0.0)
5	8 (27.6)	9 (31.0)	3 (10.3)	5 (17.2)	3 (10.3)	1 (3.4)	0 (0.0)	0 (0.0)	0 (0.0)	0 (0.0)	0 (0.0)
6	10 (47.6)	2 (9.5)	4 (19.0)	2 (9.5)	1 (4.8)	2 (9.5)	0 (0.0)	0 (0.0)	0 (0.0)	0 (0.0)	0 (0.0)
7	16 (50.0)	8 (25.0)	3 (9.4)	3 (9.4)	0 (0.0)	1 (3.1)	0 (0.0)	1 (3.1)	0 (0.0)	0 (0.0)	0 (0.0)
8	10 (27.8)	17 (19.4)	5 (13.9)	5 (13.9)	3 (8.3)	2 (5.6)	2 (5.6)	1 (2.8)	1 (2.8)	0 (0.0)	0 (0.0)
9	4 (20.0)	4 (20.0)	5 (25.0)	2 (10.0)	1 (5.0)	0 (0.0)	2 (10.0)	1 (5.0)	1 (5.0)	0 (0.0)	0 (0.0)
10	0 (0.0)	0 (0.0)	1 (25.0)	0 (0.0)	0 (0.0)	1 (2.5)	0 (0.0)	0 (0.0)	1 (25.0)	0 (0.0)	1 (25.0)
**Patients that did not achieve clinical remission (*N* = 183) – UNRS score shift from induction baseline to week 52 for mirikizumab treated patients**											
0	3 (100)	0 (0.0)	0 (0.0)	0 (0.0)	0 (0.0)	0 (0.0)	0 (0.0)	0 (0.0)	0 (0.0)	0 (0.0)	0 (0.0)
1	1 (25.0)	1 (25.0)	1 (25.0)	0 (0.0)	0 (0.0)	1 (25.0)	0 (0.0)	0 (0.0)	0 (0.0)	0 (0.0)	0 (0.0)
2	3 (25.0)	4 (33.3)	1 (8.3)	4 (33.3)	0 (0.0)	0 (0.0)	0 (0.0)	0 (0.0)	0 (0.0)	0 (0.0)	0 (0.0)
3	6 (54.5)	2 (18.2)	0 (0.0)	2 (18.2)	0 (0.0)	0 (0.0)	1 (9.1)	0 (0.0)	0 (0.0)	0 (0.0)	0 (0.0)
4	5 (23.8)	4 (19.0)	3 (14.3)	6 (28.6)	2 (9.5)	1 (4.8)	0 (0.0)	0 (0.0)	0 (0.0)	0 (0.0)	0 (0.0)
5	4 (17.4)	7 (30.4)	3 (13.0)	5 (21.7)	0 (0.0)	3 (13.0)	0 (0.0)	0 (0.0)	1 (4.3)	0 (0.0)	0 (0.0)
6	4 (13.8)	4 (13.8)	5 (17.2)	5 (17.2)	4 (13.8)	1 (3.4)	4 (13.8)	2 (6.9)	0 (0.0)	0 (0.0)	0 (0.0)
7	5 (16.1)	2 (6.5)	1 (3.2)	12 (38.9)	3 (9.7)	2 (6.5)	1 (3.2)	4 (12.9)	1 (3.2)	0 (0.0)	0 (0.0)
8	4 (11.8)	3 (8.8)	3 (8.8)	8 (23.5)	2 (5.9)	2 (5.9)	4 (11.8)	2 (5.9)	4 (11.8)	1 (2.9)	1 (2.9)
9	1 (9.1)	0 (0.0)	0 (0.0)	2 (18.2)	0 (0.0)	1 (9.1)	1 (9.1)	0 (0.0)	3 (27.3)	3 (27.3)	0 (0.0)
10	1 (25.0)	0 (0.0)	1 (25.0)	0 (0.0)	0 (0.0)	0 (0.0)	0 (0.0)	1 (25.0)	0 (0.0)	1 (25.0)	0 (0.0)

(B) Placebo-treated patients											
Baseline Urgency NRS score	Week 52 Urgency NRS score, *n* (%)										
	0	1	2	3	4	5	6	7	8	9	10
**Patients that achieved clinical remission (*N* = 45) – UNRS score shift from induction baseline to week 52 for placebo-treated patients**											
0	0 (0.0)	0 (0.0)	0 (0.0)	0 (0.0)	0 (0.0)	0 (0.0)	0 (0.0)	0 (0.0)	0 (0.0)	0 (0.0)	0 (0.0)
1	0 (0.0)	0 (0.0)	0 (0.0)	0 (0.0)	0 (0.0)	0 (0.0)	0 (0.0)	0 (0.0)	0 (0.0)	0 (0.0)	0 (0.0)
2	1 (100)	0 (0.0)	0 (0.0)	0 (0.0)	0 (0.0)	0 (0.0)	0 (0.0)	0 (0.0)	0 (0.0)	0 (0.0)	0 (0.0)
3	1 (50.0)	0 (0.0)	0 (0.0)	1 (50.0)	0 (0.0)	0 (0.0)	0 (0.0)	0 (0.0)	0 (0.0)	0 (0.0)	0 (0.0)
4	2 (66.7)	0 (0.0)	0 (0.0)	0 (0.0)	0 (0.0)	0 (0.0)	0 (0.0)	1 (33.3)	0 (0.0)	0 (0.0)	0 (0.0)
5	8 (72.7)	2 (18.2)	0 (0.0)	0 (0.0)	1 (9.1)	0 (0.0)	0 (0.0)	0 (0.0)	0 (0.0)	0 (0.0	0 (0.0)
6	3 (42.9)	0 (0.0)	4 (57.1)	0 (0.0)	0 (0.0)	0 (0.0)	0 (0.0)	0 (0.0)	0 (0.0)	0 (0.0)	0 (0.0)
7	3 (42.9)	0 (0.0)	4 (57.1)	0 (0.0)	0 (0.0)	0 (0.0)	0 (0.0)	0 (0.0)	0 (0.0)	0 (0.0)	0 (0.0)
8	2 (22.2)	5 (55.6)	2 (22.2)	0 (0.0)	0 (0.0)	0 (0.0)	0 (0.0)	0 (0.0)	0 (0.0)	0 (0.0)	0 (0.0)
9	0 (0.0)	3 (60.0)	1 (20.0)	0 (0.0)	1 (20.0)	0 (0.0)	0 (0.0)	0 (0.0)	0 (0.0)	0 (0.0)	0 (0.0)
10	0 (0.0)	0 (0.0)	0 (0.0)	0 (0.0)	0 (0.0)	0 (0.0)	0 (0.0)	0 (0.0)	0 (0.0)	0 (0.0)	0 (0.0)
**Patients that did not achieve clinical remission (*N* = 134) – UNRS score shift from induction baseline to week 52 for placebo-treated patients**											
0	0 (0.0)	0 (0.0)	0 (0.0)	0 (0.0)	0 (0.0)	0 (0.0)	0 (0.0)	0 (0.0)	0 (0.0)	0 (0.0)	0 (0.0)
1	1 (50.0)	0 (0.0)	0 (0.0)	0 (0.0)	0 (0.0)	0 (0.0)	0 (0.0)	0 (0.0)	0 (0.0)	0 (0.0)	1 (50.0)
2	0 (0.0)	1 (25.0)	1 (25.0)	0 (0.0)	1 (25.0)	0 (0.0)	0 (0.0)	1 (25.0)	0 (0.0)	0 (0.0)	0 (0.0)
3	0 (0.0)	0 (0.0)	1 (16.7)	2 (3.3)	2 (33.3)	0 (0.0)	1 (16.7)	0 (0.0)	0 (0.0)	0 (0.0)	0 (0.0)
4	0 (0.0)	2 (15.4)	5 (38.5)	1 (7.7)	3 (23.1)	1 (7.7)	0 (0.0)	1 (7.7)	0 (0.0)	0 (0.0)	0 (0.0)
5	2 (9.1)	1 (4.5)	1 (4.5)	3 (13.6)	2 (9.1)	10 (45.5)	2 (9.1)	1 (4.5)	0 (0.0)	0 (0.0)	0 (0.0)
6	3 (14.3)	1 (4.8)	3 (14.3)	4 (19.0)	2 (9.5)	2 (9.5)	1 (4.8)	2 (9.5)	2 (9.5)	0 (0.0)	1 (4.8)
7	2 (7.1)	3 (10.7)	3 (10.7)	2 (7.1)	2 (7.1)	3 (10.7)	3 (10.7)	4 (14.3)	3 (10.7)	3 (10.7)	0 (0.0)
8	1 (4.0)	2 (8.0)	0 (0.0)	1 (4.0)	2 (8.0)	3 (12.0)	4 (16.0)	2 (8.0)	6 (24.0)	3 (12.0)	1 (4.0)
9	0 (0.0)	1 (11.1)	1 (11.1)	0 (0.0)	1 (11.1)	2 (22.2)	1 (11.1)	0 (0.0)	0 (0.0)	3 (33.3)	0 (0.0)
10	0 (0.0)	0 (0.0)	0 (0.0)	1 (25.0)	0 (0.0)	0 (0.0)	0 (0.0)	1 (25.0)	0 (0.0)	1 (25.0)	1 (25.0)

Shaded cells indicate the following: purple = improved; gray = unchanged; and orange = worsened. Bolded lines denote the Urgency NRS score categories: 0–3, 4–6, and 7–10. Missing data were handled with nonresponder imputation.

Abbreviations: *N*, number of patients in the analysis population; *n*, number of patients within analyzed group achieving the endpoint of interest; NRS, Numeric Rating Scale; UNRS, Urgency Numeric Rating Scale.

Supplementary Figure 1. Patient disposition.

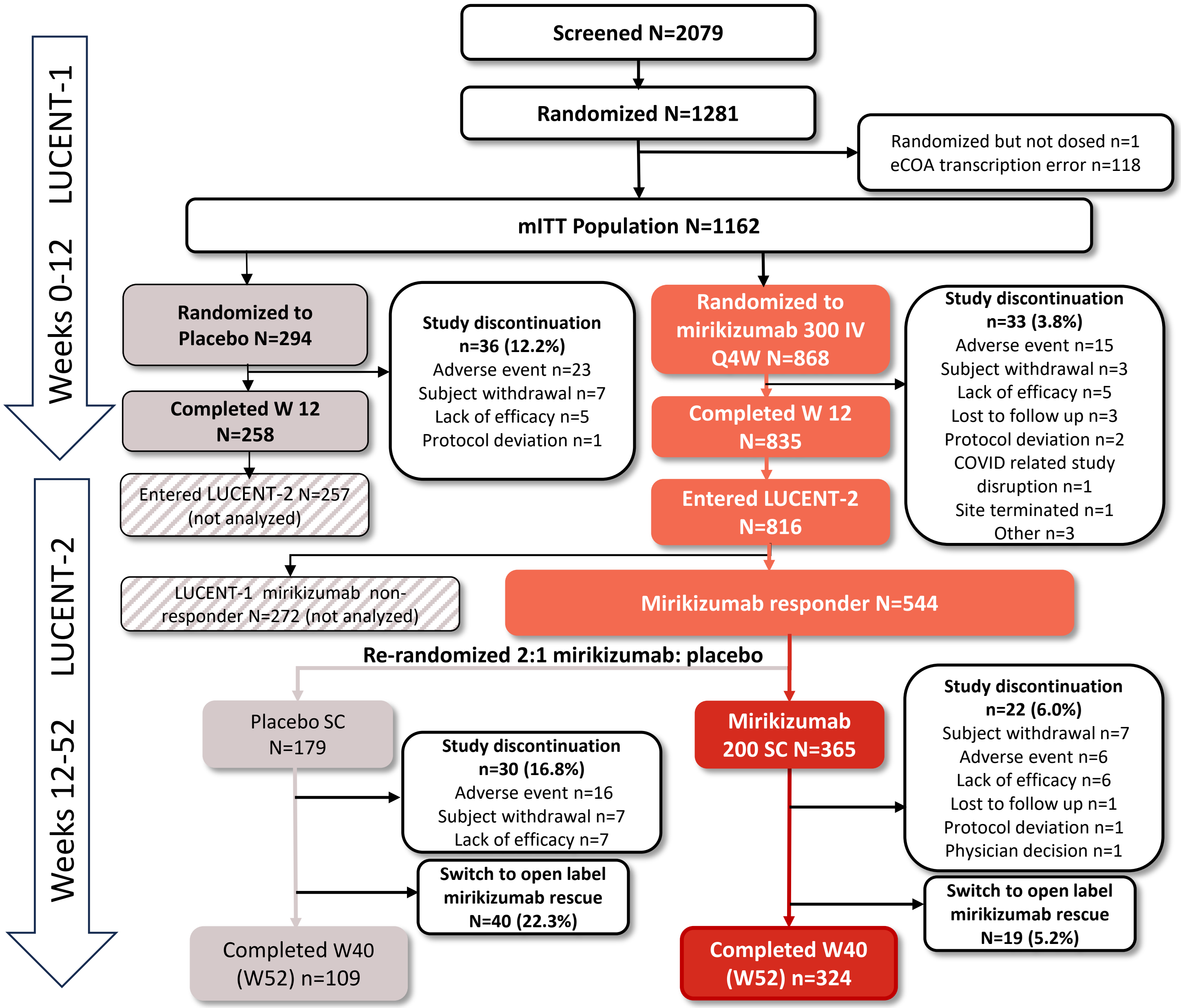

For 12-week data, mirikizumab patients were compared to placebo patients at the end of the induction study LUCENT-1. For week 52, data are based on mirikizumab induction responders in LUCENT-1 rerandomized to mirikizumab maintenance therapy or placebo in LUCENT-2. A full patient disposition figure for the studies has been published previously.[20] Abbreviations: eCOA, electronic Clinical Outcome Assessments; IV, intravenous; mITT, modified intent to

treat; SC, subcutaneous; W, week; Q4W, every 4 weeks; *N*, number of patients in the analysis population; *n*, number of patients within analyzed group achieving the endpoint of interest.

Supplementary Figure 2. Urgency NRS scores at LUCENT-1 induction baseline (week 0) for induction responders in LUCENT-2.

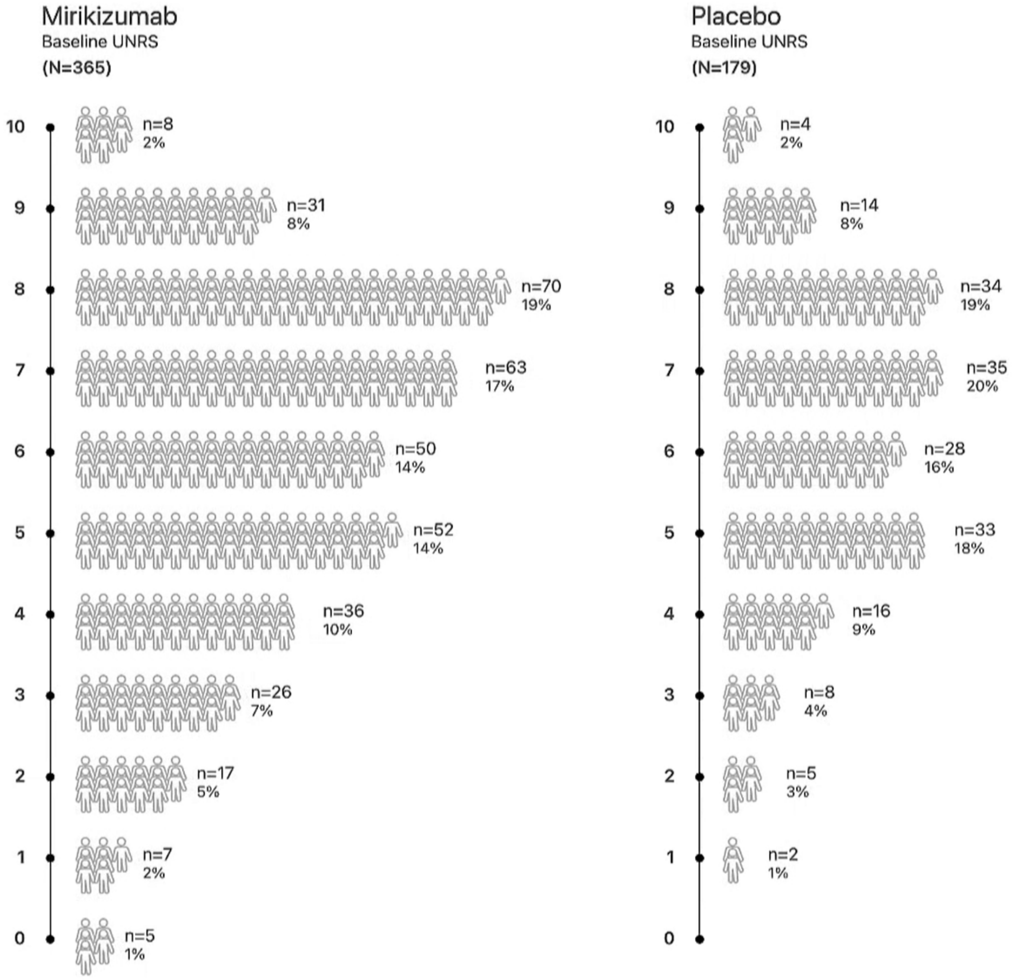

Baseline Urgency NRS scores are based on Urgency NRS scores of patients at baseline (week 0) in the LUCENT-1 induction study. Abbreviations: *N*, number of patients in the analysis population; *n*, number of patients within analyzed group achieving the endpoint of interest; NRS, Numeric Rating Scale; UNRS, Urgency Numeric Rating Scale.

Supplementary Figure 3. Shift in Urgency NRS scores from induction baseline to week 52 by treatment and clinical remission status with each baseline Urgency NRS score shift visualized: (A) mirikizumab clinical remission achieved (N=182); (B) mirikizumab clinical remission not achieved (N=183); (C) placebo clinical remission achieved (N=45); (D) placebo clinical remission not achieved (N=134).

Purple indicates improvement, gray indicates no change, and orange indicates worsening. An Urgency NRS score of 0 or 1 is considered bowel urgency remission and is noted with a black outline. Patients at week 52 were responders to mirikizumab induction therapy at week 12 who were rerandomized to mirikizumab or placebo (treatment withdrawal). Abbreviations: *N*, number of patients in the analysis population; *n*, number of patients within analyzed group achieving the endpoint of interest; NRS, Numeric Rating Scale; UNRS, Urgency Numeric Rating Scale.

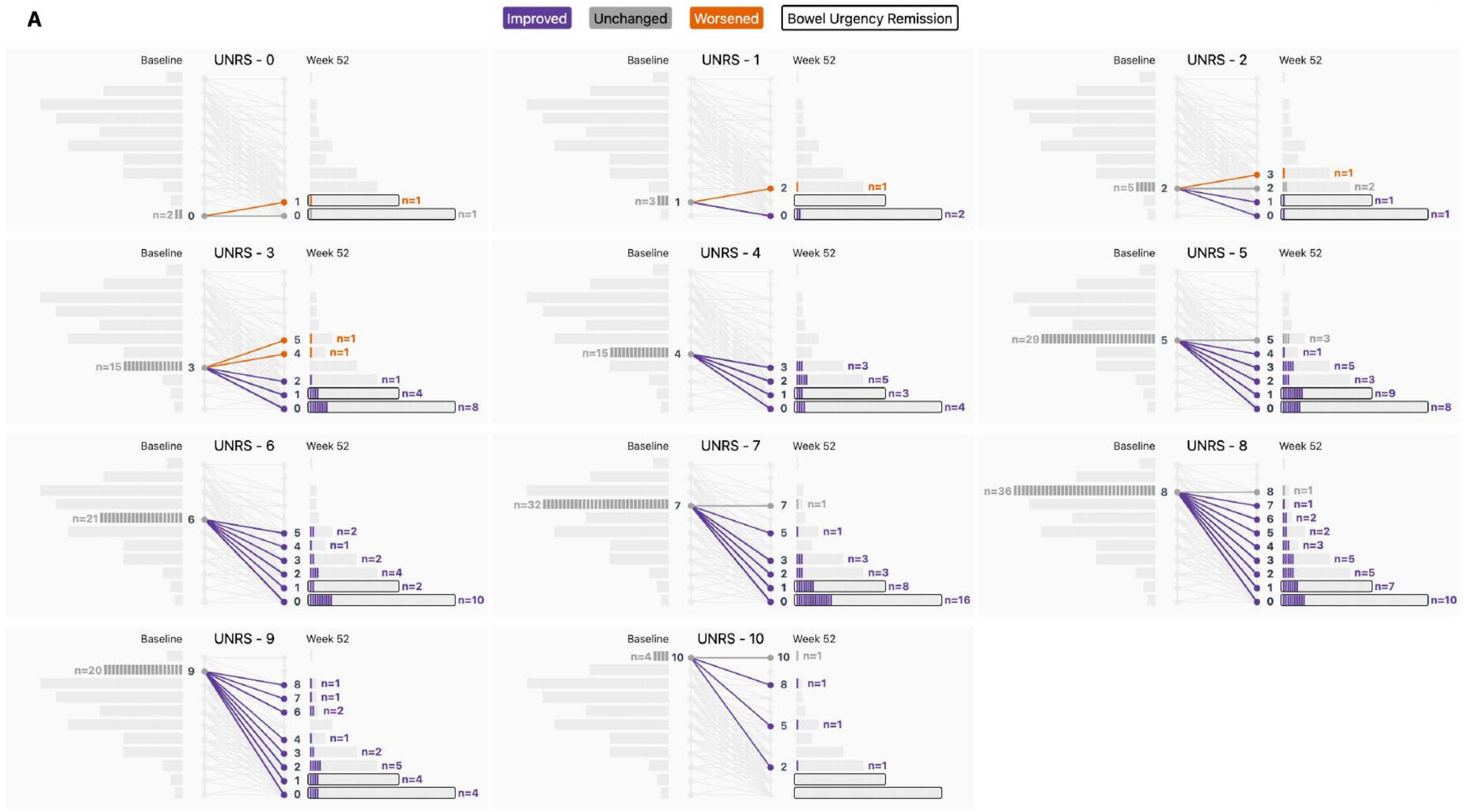
A
Improved
Unchanged
Worsened
Bowel Urgency Remission
Baseline
UNRS - 0
Week 52
UNRS - 1
UNRS - 2
UNRS - 3
UNRS - 4
UNRS - 5
UNRS - 6
UNRS - 7
UNRS - 8
UNRS - 9
UNRS - 10

B

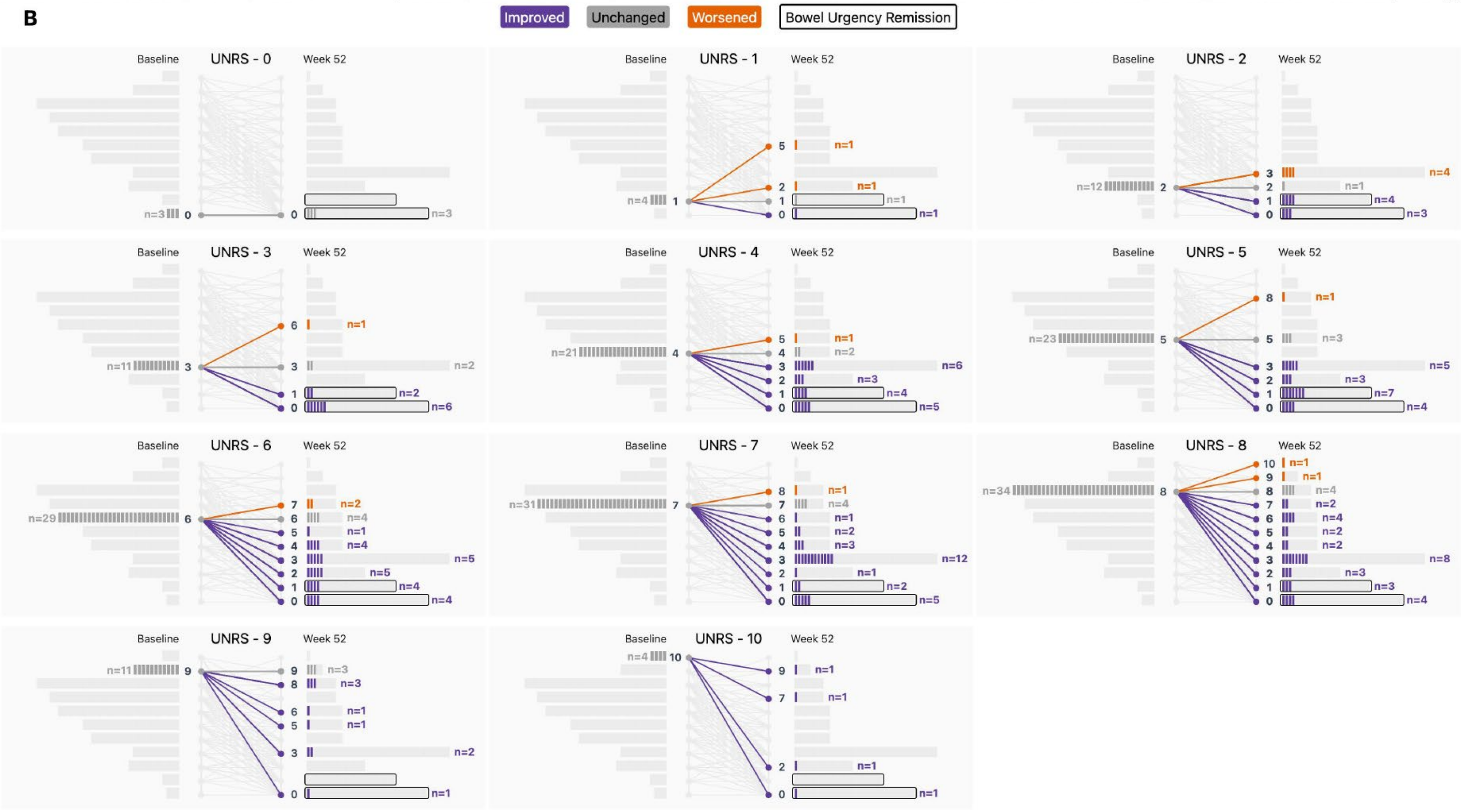
Improved
Unchanged
Worsened
Bowel Urgency Remission
Baseline
Week 52
UNRS - 0
UNRS - 1
UNRS - 2
UNRS - 3
UNRS - 4
UNRS - 5
UNRS - 6
UNRS - 7
UNRS - 8
UNRS - 9
UNRS - 10

c

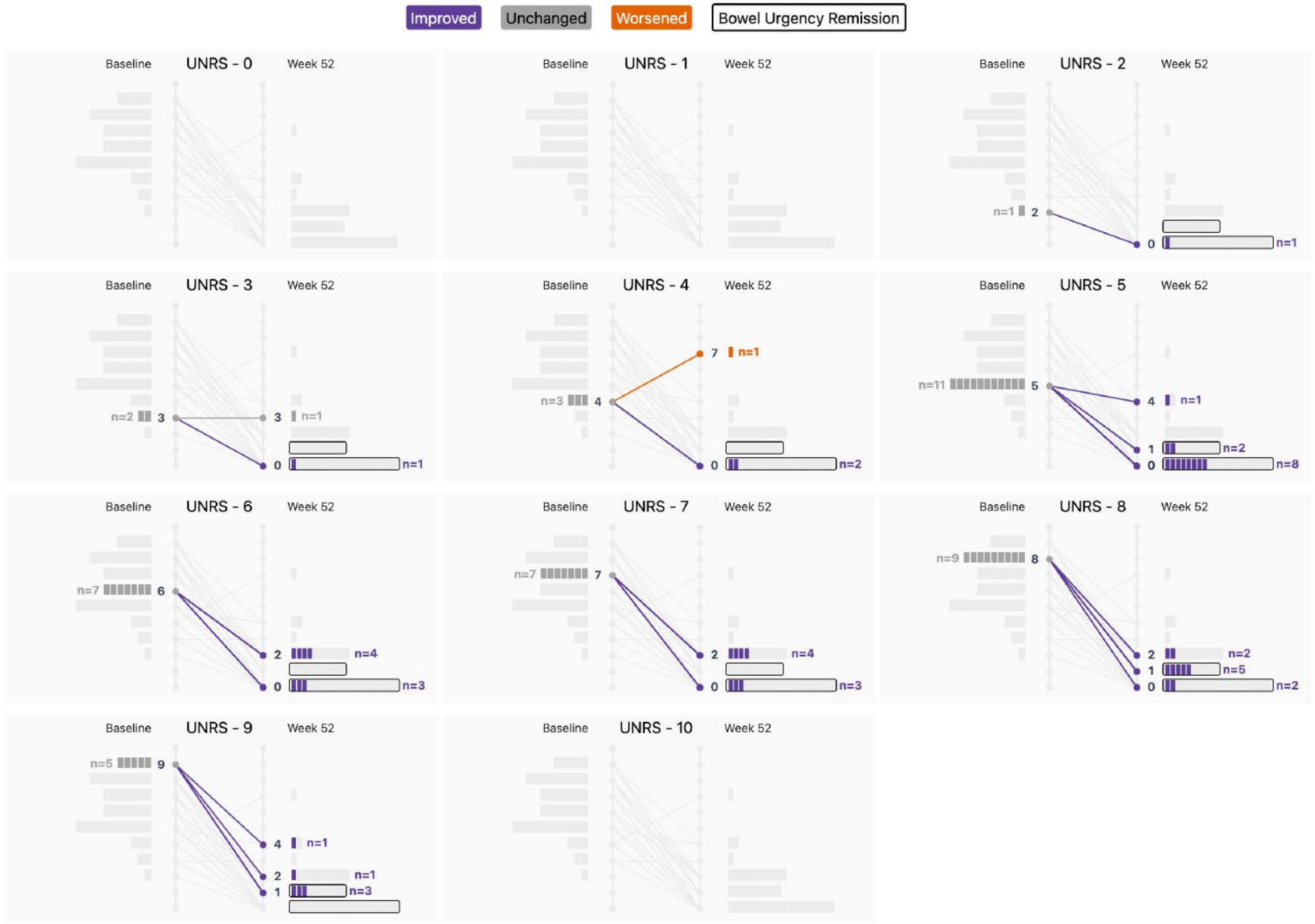
Improved
Unchanged
Worsened
Bowel Urgency Remission
Baseline
UNRS - 0
Week 52
Baseline
UNRS - 1
Week 52
Baseline
UNRS - 2
Week 52
n=1 2
0 n=1
Baseline
UNRS - 3
Week 52
n=2 3
3 n=1
0 n=1
Baseline
UNRS - 4
Week 52
7 n=1
n=3 4
0 n=2
Baseline
UNRS - 5
Week 52
n=11 5
4 n=1
1 n=2
0 n=8
Baseline
UNRS - 6
Week 52
n=7 6
2 n=4
0 n=3
Baseline
UNRS - 7
Week 52
n=7 7
2 n=4
0 n=3
Baseline
UNRS - 8
Week 52
n=9 8
2 n=2
1 n=5
0 n=2
Baseline
UNRS - 9
Week 52
n=5 9
4 n=1
2 n=1
1 n=3
Baseline
UNRS - 10
Week 52

D

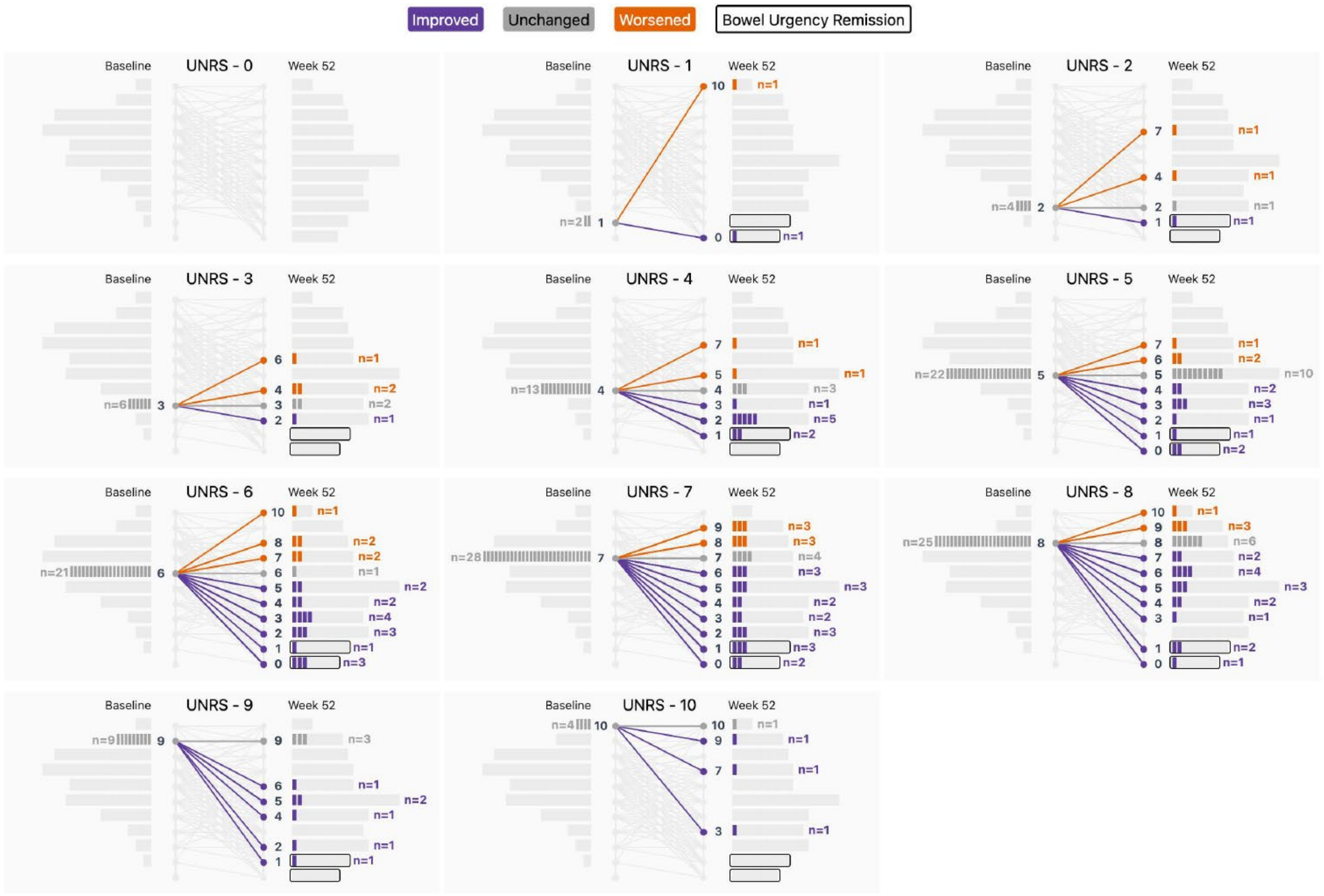
Improved
Unchanged
Worsened
Bowel Urgency Remission
UNRS - 0
UNRS - 1
UNRS - 2
UNRS - 3
UNRS - 4
UNRS - 5
UNRS - 6
UNRS - 7
UNRS - 8
UNRS - 9
UNRS - 10
Baseline
Week 52

Supplementary Figure 4. Single-point shift in Urgency NRS score from six at induction baseline to week 52 for mirikizumab-treated patients who achieved clinical remission at week 52. Purple color indicates any improvement; orange, any worsening; gray, no change.

A

BL UNRS	LUCENT-2 Week 52 UNRS, n(%) 0	1	2	3	4	5	6	7	8	9	10
0											
1											
2											
3											
4											
5											
6	10 (47.6)	2 (9.5)	4 (19.0)	2 (9.5)	1 (4.8)	2 (9.5)	0 (0.0)	0 (0.0)	0 (0.0)	0 (0.0)	0 (0.0)
7											
8											
9											
10											

**9.5%**	**Shifted to 5 = 1-pt improvement**	Clinically important to patients [a]
**4.8%**	**Shifted to 4 = 2-pt improvement**	
**9.5%**	**Shifted to 3 = 3-pt improvement**	Clinically meaningful improvement [b,c]
**19.0%**	**Shifted to 2 = 4-pt improvement**	
**9.5%**	**Shifted to 1 = 5-pt improvement**	
**47.6%**	**Shifted to 0 = 6-pt improvement**	Bowel urgency remission [b,c]

Understanding a patient's severity improvement is relevant since a 1-2-point change in UNRS score can be important to patients[a]

B

BL UNRS	LUCENT-2 Week 52 UNRS, n(%) 0	1	2	3	4	5	6	7	8	9	10
Miri-treated patients – Achieved Clinical Remission											
6	10 (47.6)	2 (9.5)	4 (19.0)	2 (9.5)	1 (4.8)	2 (9.5)	0 (0.0)	0 (0.0)	0 (0.0)	0 (0.0)	0 (0.0)
Miri-treated patients – Did Not Achieve Clinical Remission											
6	4 (13.8)	4 (13.8)	5 (17.2)	5 (17.2)	4 (13.8)	1 (3.4)	4 (13.8)	2 (6.9)	0 (0.0)	0 (0.0)	0 (0.0)
PBO-treated patients – Achieved Clinical Remission											
6	3 (42.9)	0 (0.0)	4 (57.1)	0 (0.0)	0 (0.0)	0 (0.0)	0 (0.0)	0 (0.0)	0 (0.0)	0 (0.0)	0 (0.0)
PBO-treated patients – Did Not Achieve Clinical Remission											
6	3 (14.3)	1 (4.8)	3 (14.3)	4 (19.0)	2 (9.5)	2 (9.5)	1 (4.8)	2 (9.5)	2 (9.5)	0 (0.0)	1 (4.8)

Greater UNRS improvement shifts…

with MIRI compared with PBO

with MIRI and PBO when clinical endpoint achieved compared with not achieved

[a]Dubinsky MC, et al. J Patient Rep Outcomes. 2022;6:31.

[b]Dubinsky MC, et al. J Patient Rep Outcomes. 2022;6:114.

[c]Dubinsky MC, et al. Crohns Colitis 360. 2022;5:1–13.

Abbreviations: BL, baseline; MIRI, mirikizumab; N, number of patients in the analysis population; NRS, Numeric Rating Scale; PBO, placebo; pt, point; UNRS, Urgency Numeric Rating Scale.

Supplementary Figure 5: Five factors supporting the clinical relevance of assessing bowel urgency severity over time

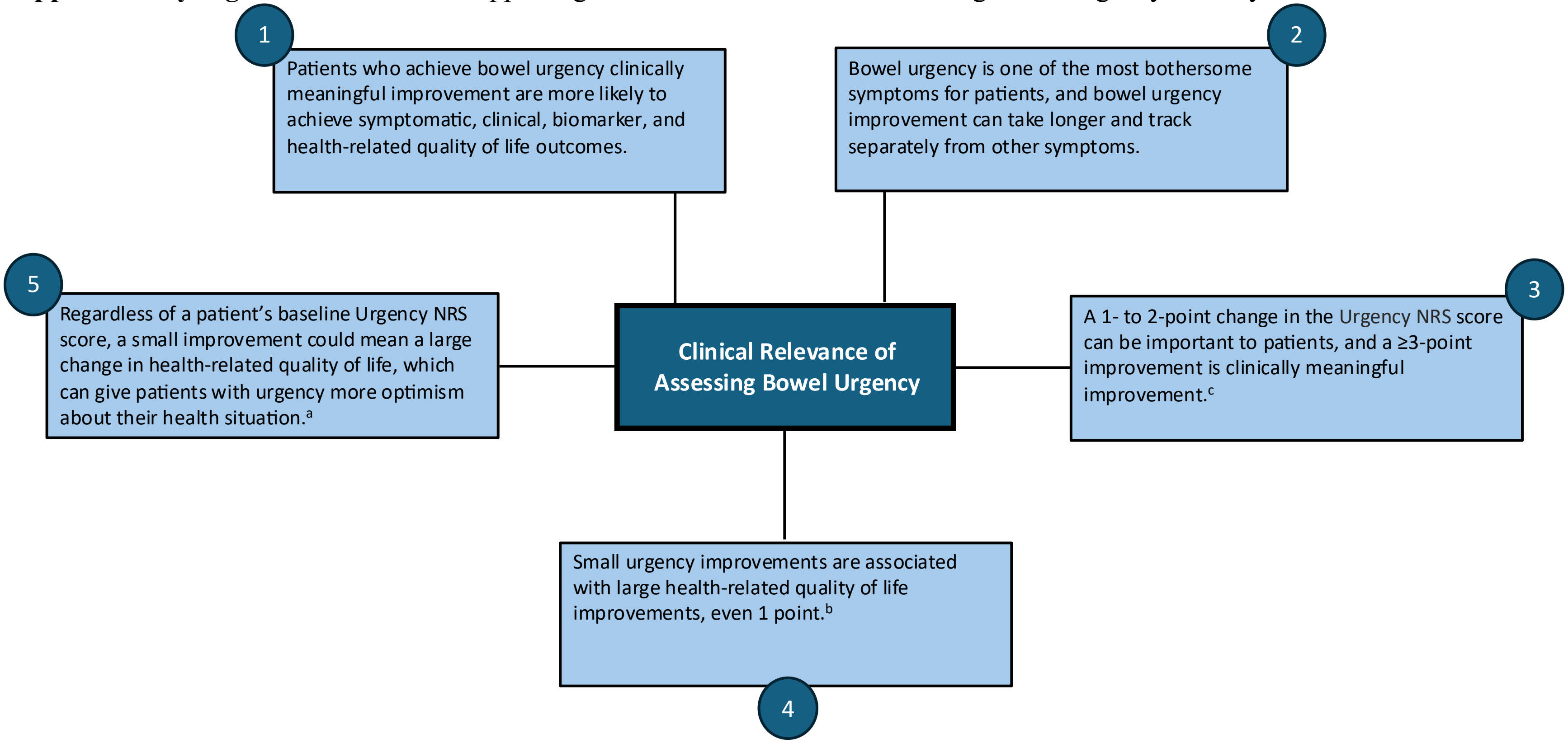

[a]Patient response trajectory analyses have identified different mirikizumab patient response patterns that have been called Super Responders, Responders, Delayed Responders, and Non/Incomplete Responders. Week 8 bowel urgency clinically meaningful improvement is a marker of being a mirikizumab Super Responder.

[b]Each additional 1-point reduction in the Urgency NRS score corresponds to improvements in patient-reported outcomes of Fatigue Numeric Rating Scale, Inflammatory Bowel Disease Questionnaire, short-form-36 Mental Component and Physical Component Scores, and the Work Productivity Activity Impairment Productivity assessment.

[c]Patients who experience improvement in bowel urgency during mirikizumab therapy may show a 1- to 10-point reduction on the Urgency NRS, depending on their baseline score. Most patients achieve clinically meaningful improvements, with a substantial proportion reaching bowel urgency remission. Among mirikizumab responders, improvements were distributed across the full range of possible scores, with some showing only modest changes of 1, 2, or 3 points. Even small reductions in Urgency NRS are clinically relevant, regardless of whether the score remains above the remission threshold of 0 or 1.
